# Polyglycerol Hyperbranched Polyesters: Synthesis, Properties and Pharmaceutical and Biomedical Applications

**DOI:** 10.3390/ijms20246210

**Published:** 2019-12-09

**Authors:** Alexandra Zamboulis, Eirini A. Nakiou, Evi Christodoulou, Dimitrios N. Bikiaris, Eleana Kontonasaki, Liliana Liverani, Aldo R. Boccaccini

**Affiliations:** 1 Laboratory of Polymer Chemistry & Technology, Department of Chemistry, Aristotle University of Thessaloniki, 54124 Thessaloniki, Greece; azampouli@chem.auth.gr (A.Z.); renia.nakiou@hotmail.com (E.A.N.); evicius@gmail.com (E.C.); 2 Department of Dentistry, Aristotle University of Thessaloniki, 54124 Thessaloniki, Greece; kont@dent.auth.gr; 3 Institute of Biomaterials, Department of Material Science and Engineering, University of Erlangen-Nuremberg, Cauerstr. 6, 91058 Erlangen, Germany; liliana.liverani@fau.de

**Keywords:** polyglycerol polyesters, hyperbranched polymers, catalysts, esterification reactions, polycondensation, medical and biomedical applications

## Abstract

In a century when environmental pollution is a major issue, polymers issued from bio-based monomers have gained important interest, as they are expected to be environment-friendly, and biocompatible, with non-toxic degradation products. In parallel, hyperbranched polymers have emerged as an easily accessible alternative to dendrimers with numerous potential applications. Glycerol (Gly) is a natural, low-cost, trifunctional monomer, with a production expected to grow significantly, and thus an excellent candidate for the synthesis of hyperbranched polyesters for pharmaceutical and biomedical applications. In the present article, we review the synthesis, properties, and applications of glycerol polyesters of aliphatic dicarboxylic acids (from succinic to sebacic acids) as well as the copolymers of glycerol or hyperbranched polyglycerol with poly(lactic acid) and poly(ε-caprolactone). Emphasis was given to summarize the synthetic procedures (monomer molar ratio, used catalysts, temperatures, etc.,) and their effect on the molecular weight, solubility, and thermal and mechanical properties of the prepared hyperbranched polymers. Their applications in pharmaceutical technology as drug carries and in biomedical applications focusing on regenerative medicine are highlighted.

## 1. Introduction

Aliphatic polyesters are widely used for biomedical, pharmaceutical, and environmental applications due to their inherent hydrolytic instability, high biodegradability and low cost of production [1]. They can be synthesized in different ways, from a broad variety of monomers and with a wide range of properties by melt polycondensation or ring-opening-polymerization (ROP). The most studied and used aliphatic polyesters are poly(lactic acid) (PLA), poly(glycolic acid) (PGA), their copolymer poly(lactic-co-glycolic acid) (PLGA), poly(hydroxyl alkanoates) (PHA), and poly(caprolactone) (PCL). However, these polymers have some limitations. Their hydrophobicity for example. Additionally, despite their inherent biodegradability, some are characterized by a slow degradation rate. It is generally accepted that for biomedical applications, a short chain in between the two ester groups is a prerequisite for a hydrolysis within a reasonable time frame [2]. 

Polyesters from bio-based monomers are expected to guarantee a low toxicity and good biocompatibility and are thus attracting a renewed interest. In parallel, hyperbranched polymers consist an emerging novel class of polymers with considerable potential [3]. Hyperbranched polymers are related to dendrimers, a family of highly branched monodispersed macromolecules. Both macromolecular structures bear branch points, but while dendrimers have a regular and cascade-like structure, hyperbranched polymers exhibit random branching, with higher polydispersities and some architectural defects. In spite of their advantages, dendrimers are produced by elaborate multi-step syntheses and typically demand tedious purifications. Due to the high cationic charge density some of them usually bear, dendrimers interact with biological cell membranes, resulting in undesired membrane disruption. Finally, some dendrimers, such as polyamidoamine (PAMAM) and polyethylenimine (PEI) for example, can be hydrolyzed in vivo to monomers which may cause some toxicity. On the other hand, hyperbranched polymers produced from aliphatic acids do not present such problems: indeed, they are completely biocompatible materials and can be hydrolyzed to non-toxic monomers. Additionally, they can be easily prepared by a one-step reaction and are expected to be produced at a lower cost. Various strategies have been employed to synthesize hyperbranched polymers. The most common route is the condensation of A_x_B monomers. As it will be detailed further on, hyperbranched glycrerol polyesters are generally synthesized by the reaction of A_3_ (glycerol) with B_2_ (diacid) monomers. Alternatively, multifunctional cores have been used as branching starting points. 

Glycerol is the simplest triol, it is a clear, viscous, hygroscopic liquid at room temperature (melting point 17.8 °C, boiling point 290.0 °C) and a prime example of a bio-based monomer. It is naturally synthesized by diverse biological pathways and can be metabolized back to glucose through a series of chemical transformations in the liver. Since glycerol is a trifunctional monomer, it can be used to synthesize hyperbranched polymers. Additionally, glycerol, which is largely produced as the main by-product of the transesterification process of biodiesel production, is recognized according to U.S. Department of Energy as one of the most important building blocks or top value-added chemicals derived from biomass [4]. Therefore, hyperbranched glycerol polyesters are not only expected to be biocompatible and non-toxic, but also sustainable. The production of glycerol is expected to increase significantly in the next decades and its polymerization into value added materials is a strategy to exploit this by-product [5]. For these reasons, polymers based on glycerol and α,*ω*-carboxy diacids have gained considerable interest for the development of hyperbranched biodegradable and biocompatible materials appropriate for several applications including pharmaceutical and biomedical ones. 

**Scheme 1:**
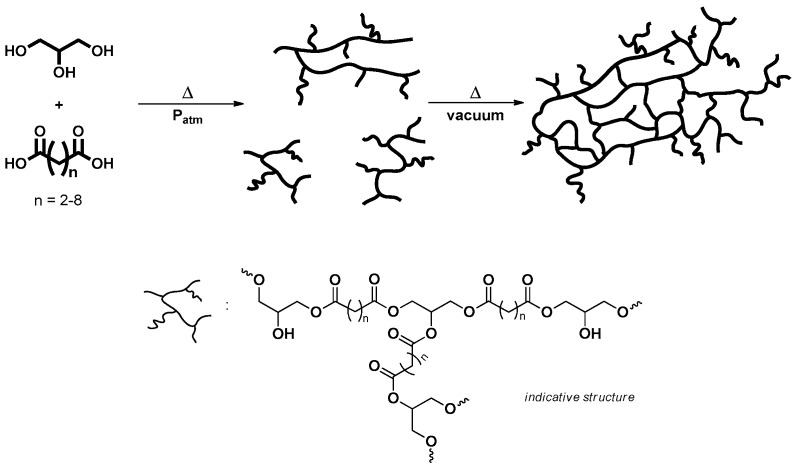
Schematic representation of the general synthesis of glycerol polyesters. Adapted with permission from [6]. Copyright 2013 American Chemical Society.

Glycerol polyesters with α,*ω*-carboxy diacids are generally synthesized according to Scheme 1, at high temperatures, with or without catalyst. The three hydroxyl groups of glycerol do not have the same reactivity. Initially, the acid reacts with the primary hydroxyl groups and to a lower extent with the secondary -OH. During the second step, due to the low concentration of primary -OH, the esterification of the secondary -OH dominates, creating crosslinks between linear chains.

Polyesters based on glycerol possess similar properties to low-molar mass glycerides, but have a higher mechanical stability and longer in vivo circulation times. Since ester bonds are hydrolytically unstable, these materials will degrade over time under physiological conditions. Their biodegradability, along with the low costs of production, are considered to be the main factors for their widespread use. 

Glycerol-based polymers are expected to render innovative materials that are useful in orthopedic and ophthalmic applications, reconstructive surgery, tissue engineering, and as drug delivery agents [7,8,9]. The spectrum of applications of a polymer is greatly broadened when functional groups are incorporated into its backbone [10]. Glycerol polyesters with pending hydroxyl groups appropriate for post-polymerization functionalization hold promises for the tuning of their properties, allowing new applications to be envisioned. 

Related to glycerol, hyperbranched polyglycerol (HBPG) has been used as a substitute for polyethylene glycol (PEG) owing to its many potential advantages [11,12]. Firstly, HBPG is more hydrophilic than PEG. Secondly, the hyperbranched structure enables HBPG to cover surfaces more efficiently than PEG. 

In the present article, we review the recent advances in the field of glycerol polyesters based on aliphatic dicarboxylic acids (from succinic to sebacic acids) and the copolymers of glycerol or HBPG with PLA and PCL, spotlighting the relations between the synthesis and the mechanical properties and degradation of the polymers. Novel copolymers and original synthetic strategies are reported along with applications in the fields of drug delivery and tissue engineering. 

## 2. Poly(Glycerol Succinate), PGSuc 

### 2.1. Synthesis and Properties

#### 2.1.1. PGSuc

**Scheme 2:**
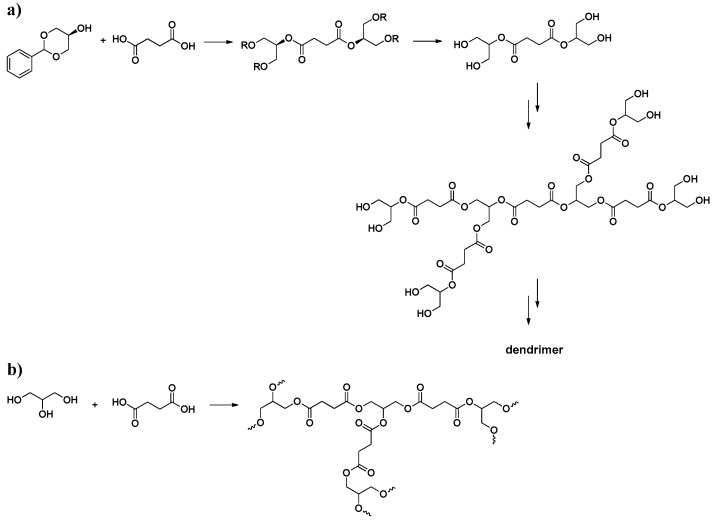
(**a**) Synthesis of PGSuc dendrimers from glycerin benzylidene acetal and succinic acid. (**b**) Synthesis of branched polymers from glycerol and succinic acid.

Succinic acid (SuA) is an important aliphatic acid bio-based monomer. It is a white, odorless solid, soluble in water, ethanol, and acetone. According to the US Food and Drug Administration (FDA) both glycerol and succinic acid are generally regarded as safe materials and have been approved for medical applications. Both these natural compounds are abundant and inexpensive starting materials, and when joined together biodendrimers can produced. Carnahan et al. reported the synthesis of PGSuc dendrimers by using benzylidene acetal as protecting group, which can be selectively removed under mild conditions (Scheme 2a) [13]. All dendrimers were found to be amorphous and their glass transition temperatures (T_g_) increased from –20 to –14 °C as the molecular weight increased. However, this procedure was complicated and required many steps. In a simpler way, branched oligoesters, based on glycerol and succinic acid, with various controllable sizes and topologies were prepared by Agach et al. without the use of solvent nor catalyst, according to Scheme 2b [14]. 

According to literature models, the gelation of the present system should occur in a range of Gly/SuA molar ratios of 0.33–1.33 at full conversion of the reactant in default [15]. For this reason, Agach et al. employed higher molar ratios (i.e., 1.3, 1.5, 1.7, and 1.9) [14]. Polymerization was carried out at 190 °C under constant mechanical stirring and atmospheric pressure for 24 h. The reaction progress was followed by end groups measurements and it was observed that while –COOH were almost converted after 12 h, the amount of unreacted –OH increased at higher Gly/SuA molar ratios. The results obtained from size exclusion chromatography clearly fit the theory: the smaller the Gly/SuA molar ratio, the bigger the number average degree of polymerization, the number average molecular weight (M_n_), the degree of branching (DB) and as a result, the higher the average number of dendritic units per molecule (1.5 > 1.7 > 1.9), Table 1. These PGSuc oligomers were found to be non-ecotoxic, readily biodegradable and easily chemically degradable. Hydrolysis depended on the monomer molar ratio of oligoesters (1.3 > 1.5 > 1.7 > 1.9), the used pH (4, 6, or 9) and the temperature.

In a similar procedure PGSuc was synthesized by a melt polycondensation procedure at 180 °C but using different Gly/SuA molar ratios (0.6:1, 0.9:1, and 1.2:1) and reaction times, again without the addition of a solvent or a catalyst [16]. Even though the aim was not to study the result of the reaction parameters on the produced polymer, it was reported that the weight average molecular weight (M_w_) decreased by increasing the molar ratio of monomers, which is in good agreement with the study of Agach et al. [14], and that all polyesters were amorphous with T_g_ ranging between −15.2 and −29.9 °C (Table 1). 

In a recent work of Valerio et al. the synthesis of PGSuc polyesters using glycerol of different purities and sources was described in order to evaluate the effect of glycerol purity on polyesters properties [17]. Polymerization was carried out at 180 °C using different monomer ratios and reaction times. Amorphous polyesters produced with pure glycerol gave PGSuc with the lowest T_g_ (−10.9 °C) and T_g_ increased with decreasing glycerol purity (Table 1). Reaction temperatures of 180–190 °C are quite high and some highly branched polyesters, leading to insoluble gels, were obtained. To avoid this PGSuc was also synthesized at lower temperatures (indicative conditions: 135 °C for 24 h) with toluene and 1:1 or 2:1 diacid:glycerol molar ratio [18]. Compared to poly(glycerol azelate) and poly(glycerol glutarate), the reactions involving succinic acid resulted in products with higher degree of polymerization (DP). Wyatt et al. further reported the synthesis of oligomeric prepolymers by reacting succinic acid with glycerol at low temperatures but in the presence of a catalyst [19]. Both monomers in almost equimolar amounts and with 0.15% *w*/*w* Ti(OC_4_H_9_)_4_, were heated, under reduced pressure (~150 Pa), for 1 h at 60 °C, 2 h at 100 °C, 2 h at 120 °C, and finally 11 h at 150 °C. The hyperbranched prepolymers were obtained in an average yield of 62%, and gelation was not observed. The average M_w_, polydispersity index (PDI) and DP were determined by size exclusion chromatography and found to be 992, 1.28, and 3.0 g/mol respectively. 

#### 2.1.2. Synthesis of PGSuc Polyesters with Side Groups and PGSuc Copolymers

**Scheme 3:**
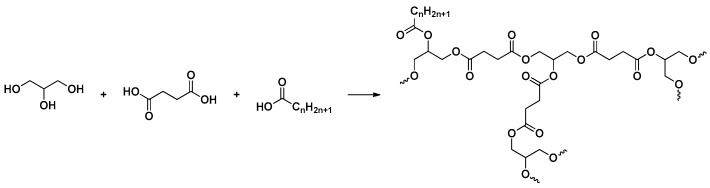
Synthesis of polymeric surfactants from glycerin, succinic acid and an aliphatic carboxylic acid.

Bio-based surfactants, with PGSuc as the polar head and acyl groups, ranging from 8 to 14 carbon atoms, as hydrophobic tails were synthesized (Scheme 3) [20,21,22]. For their synthesis, all monomers (glycerol, succinic acid and the aliphatic carboxylic acid) were added simultaneously in the reactor and esterification proceeded at 150 °C in the presence of a catalyst (sulfuric acid was used as Brønsted catalyst) or not. According to this procedure a large range of polymeric materials was designed, depending on the kind of acyl group and fatty chain/succinate molar ratios. Non-ionic PGSuc-sorbitan oligoester surfactants were also synthesized [23]. Oligoesters could form stable foams, they exhibited a wide range of wetting powers and displayed excellent micellar solubilization properties. In a similar procedure butyl glycidyl ether comonomer was used to produce a hyperbranched architecture with side reactive groups [24]. 

**Figure 1:**
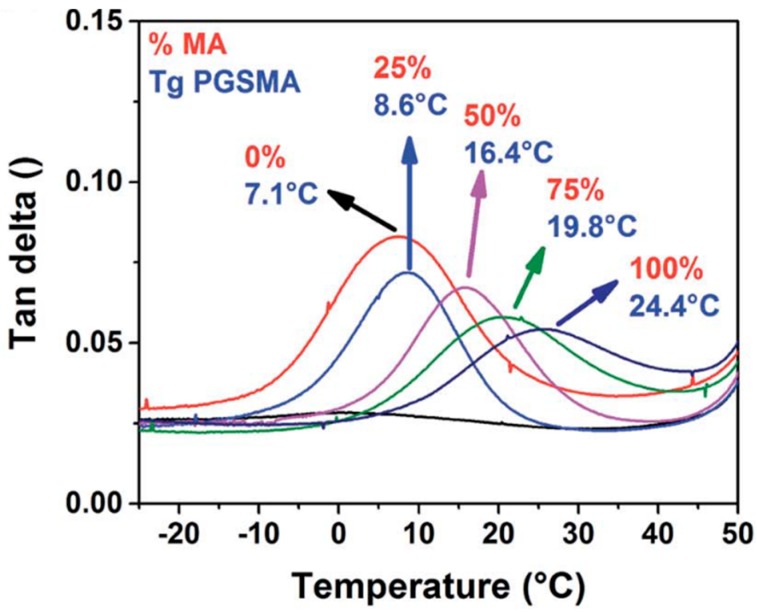
Tan delta of poly(glycerol succinate-co-maleate (PGSMA) phase of dynamically vulcanized 80/20 wt % poly(lactic acid) (PLA)/PGSMA blends. From reference [27]. Published by The Royal Society of Chemistry.

Except for neat PGSuc polyesters and polyesters with side acyl groups, copolymers from SuA with other acids have been also synthesized. Recently, Baharu et al. reported the synthesis of new elastic polyesters via the catalyst-free polyesterification of glycerol with a mixture of succinic and azelaic acid [25]. A 1:1 molar ratio acid:Gly was used, polymerization took place at 160–165 °C for 2 h, and further polyesterification of the prepolymer was carried out at 125 °C for 48 h to produce films. The T_g_ were found to range between −8 °C and −23 °C depending on the ratio of the monomers. Poly(glycerol succinate-co-maleate) (PGSMA) copolymers were synthesized by melt polycondensation at 130 °C with stannous octoate as a catalyst, using a 2:2:1 molar ratio of glycerol: succinic anhydride: maleic anhydride [26]. Addition of nanocrystalline cellulose caused an increase in the composite stiffness and ultimate tensile strength and a reduction in the elongation at break. Similar PGSMA copolymers were also used to prepare blends with PLA and poly(butylene succinate) with enhanced mechanical properties [27,28,29]. The synthesized copolymers were thermally stable materials, and dynamic mechanical analysis confirmed that the T_g_ of copolymers increased by increasing the maleic anhydride content (Figure 1). 

### 2.2. Applications

**Figure 2:**
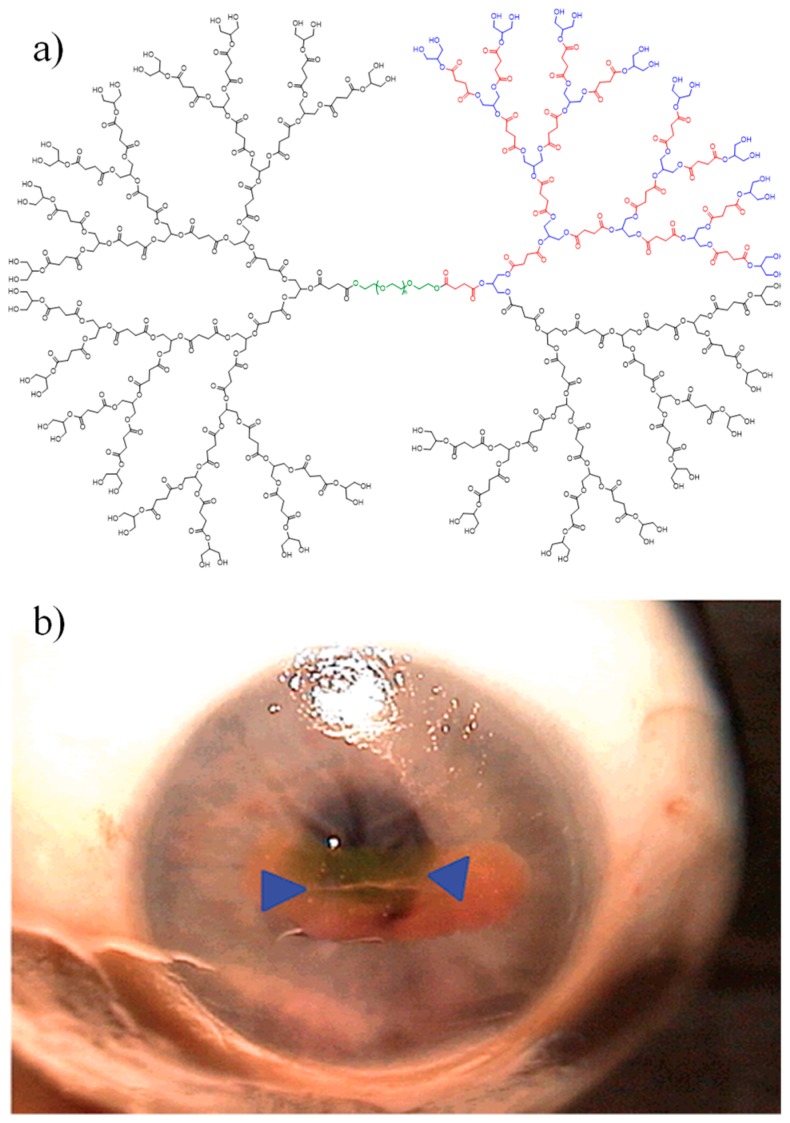
(**a**) Poly(glycerol succinate)-poly(ethylene glycol) hybrid dendritic-linear copolymer. (**b**) Optical image of an eye with a sealed 4.1 mm linear laceration using PGSuc–PEG copolymer. The sealed laceration is the horizontal line between the two arrowheads. Reprinted with permission from [32]. Copyright 2002 American Chemical Society.

As mentioned earlier, PGSuc polyesters with side acyl groups were evaluated as bio-sourced, biodegradable surfactants [20,22,23], while PGSMA was used in blend with PLA to increase its thermal and mechanical properties [26,27,28,29]. At this point, although not being strictly hyperbranched, it is noteworthy to report the work of Grinstaff et al. who explored dendritic structures based on glycerol and succinic acid [30,31,32]. Indeed, PGSuc bio-based dendritic macromolecules were found to be biocompatible and appropriate as new surgical materials for orthopedic or ophthalmic operations and the challenging field of tissue engineering [19]. In this context, Luman et al. reported the use of PGSuc dendritic structures to seal corneal wounds [30], while others have investigated their use as carriers for small molecules like drugs or dyes [31]. Novel poly(glycerol succinate)-poly(ethylene glycol) hybrid dendritic-linear copolymers composed of PEG linear chains and PGSuc dendritic blocks were synthesized using an efficient and high yield divergent procedure (Figure 2a) [32]. A small ratio between the molecular weights of the linear part and the dendritic block was chosen. The copolymers presented a great aqueous solubility. Once prepared, these dendritic macromolecules were further functionalized with photo-crosslinkable groups. The tissue adhesive properties of the gels were sufficient enough to seal corneal lacerations and the PGSuc–PEG copolymer had better performances than conventional sutures, withstanding greater pressures and stresses placed on or around the wound site (Figure 2b).

## 3. Poly(Glycerol Glutarate), PGGlu

Glutaric acid (GA) [HOOC(CH_2_)_3_COOH] has been also used for the production of aliphatic polyesters and the synthesis of hyperbranched polyesters with glycerol. However, PGGlu has not been extensively studied compared with other aliphatic diacids. A first synthesis of PGGlu was reported by Wyatt et al.: glutaric acid was dissolved in dimethylformamide (DMF) or dimethylsulfoxide (DMSO), slowly added to a solution of glycerol and titanium(IV) butoxide (catalyst) and heated at 150 °C (22 h), affording oligomers with M_n_ = 2402 g/mol [33]. In another work of the same group dibutyltin oxide was used to catalyze the synthesis of oligo(glycerol glutaric acid)s in the absence and presence of DMF [34] or toluene [35] as solvents. In neat conditions very high M_n_ polyesters were prepared (M_n_ = 445,000 g/mol) while in the presence of DMF only oligomers were obtained (M_n_ = 1058–1474 g/mol, depending on reaction time), (Table 2). The T_g_ increased with increasing DB and M_n_, ranging from −50.1 °C for the low molecular weight to −31.3 °C for the solvent free polyester. Thermal decomposition of all polyesters was relatively low, since mass loss started at about 200 °C, but high M_n_ polyesters exhibited higher stabilities. The Gly/GA molar ratio, since it affected the DB, played also an important role on the properties of the prepared materials [18]. When 1:1 and 1:2 Gly/GA molar ratios were used in toluene, it was found that the glutaric acid-derived polymers gave the highest DB (31.2%, 85.6%, respectively) after 24 h [35]. T_g_ values of −13.9 °C and −13.0 °C were reported for PGGlu prepared in toluene at 135 °C, for Gly/GA molar ratio 1:2 and 2:1 respectively [36]. The prepared hyperbranched polyesters had very low tensile strength 0.41 and 0.62 MPa, Young’s modulus 1.94 and 4.58 MPa and elongation at break 26.9% and 16.3%, respectively. Gas chromatography provided evidence that low amounts of oligomers remained in the final synthesized polyester, indicating the reaction was not complete [37]. 

The solvent and water absorption of PGGlu films have been evaluated [38,39]. Additionally, in a recent study, monoglycerides (MGs) were incorporated to PGGlu films in order to investigate their effect on the mechanical properties and the solvent absorption of films [40]. It was found that the incorporation of MGs improved the absorption properties of the films but led to a reduction of their stiffness. 

Though the synthesis and some properties of PGGlu have been studied, as described previously, to the best of our knowledge, no potential applications of such hyperbranched polyesters have yet been reported. 

## 4. Poly(Glycerol Adipate), PGAd

### 4.1. Synthesis and Properties

**Scheme 4:**
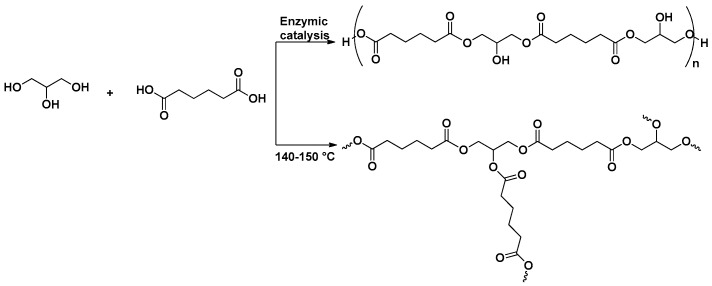
Synthesis of linear and branched poly(glycerol adipate) (PGAd), from glycerol and adipic acid.

Poly(glycerol adipate) (PGAd) is a polyester obtained from two non-toxic and bio-based monomers, glycerol and adipic acid (AdA) [41], which are both safe and FDA-approved compounds [42]. PGAd is encountered as a linear or a hyperbranched polymer, depending on the catalysis (Scheme 4). Linear PGAd bears a pendent hydroxyl group in every repeating unit that, as will be developed later, can be further functionalized. 

#### 4.1.1. Linear PGAd 

Linear PGAd has been synthesized by lipase-catalyzed polycondensation reactions. Enzymatic catalysis is in agreement with the principles of green synthesis and is receiving much attention for polymer production, since biocatalysis is expected to generate desired properties from an environmental point of view, such as biodegrability and biocompability [43]. The reaction of hydroxyl groups on a multifunctional compound such as glycerol, depends on the relative reaction rate of primary and secondary groups. Although primary hydroxyls are more reactive, protecting the secondary hydroxyl group of glycerol is necessary to obtain a linear polyester. In contrast, with enzymatic catalysis the selectivity of enzymes to the primary -OH groups of glycerol during condensation reactions is very high (90–95%) [44] due to steric hindrance, and linear polyesters are obtained without the need of protecting the pendent hydroxyl groups [10]. The most widely used lipase for the enzymatic synthesis of polyesters is Novozym 435. It is a triacyl glycerol lipase derived from *Candida antarctica* fraction B, immobilized on a macroporous acrylic resin [44]. Its success is due to its temperature and organic-solvent tolerance, and its high regio-, chemo- and stereo-selectivity [45]. 

**Scheme 5:**
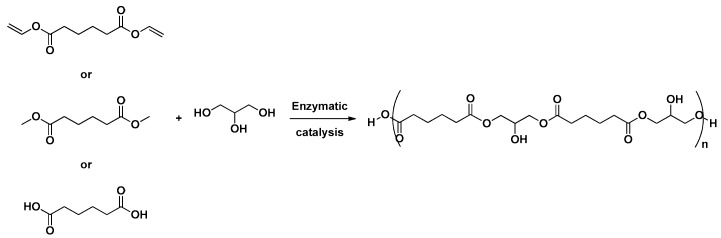
General approaches for the synthesis of linear poly(glycerol adipate)(PGAd) under enzymatic catalysis.

Three different strategies, illustrated in Scheme 5, have been applied to the enzymatic synthesis of PGAd [46]. According to the first approach, glycerol was enzymatically polymerized with divinyl adipate (DVA) in the presence of Novozym 435. Typically, equimolar amounts of divinyl adipate and glycerol were heated at 50 °C, in the presence of 1 wt % enzyme, for 24 h [44]. The polymer was then heated at 90–95 °C to deactivate any residual enzyme which might have leached out of the resin. The vinyl alcohol produced as a by-product was directly converted into acetaldehyde by tautomerization, and finally evaporated at the reaction temperature; shifting the equilibrium of the polycondensation reaction towards the products. The polymer was recovered as a clear, viscous liquid. In spite of its free hydroxyl groups, the obtained polymer is altogether insoluble in water and hydrophilic [45,47,48]. In the absence of enzyme, crosslinked products were obtained [44]. In a similar procedure, Taresco et al. studied the effect of the reaction temperature on the degree of branching and the molecular weight during polymerization of Gly and DVA at 40, 60, and 70 °C for 24 h [45]. It was observed that the degree of branching increased from 5% up to ~30% when the reaction temperature increased. Indeed, at higher temperatures, the selectivity of Novozym 435 for primary hydroxyl groups decreased, leading to more branched polymers. Due to this phenomenon the calculated average molecular weight was higher at lower temperatures (40 or 50 °C). Kline et al. reported that by increasing the reaction time the molecular weight of the polyesters increased up to 12,000 g/mol, but no branching was detected [44]. This is in good agreement with the work of Naolou et al., according to whom, no branching was observed below 60 °C, even after 9 h [49]. 

The second strategy to synthesize PGAd consists in replacing divinyl adipate by dimethyl adipate [50]. Novozym 435 has been also widely used for this polymerization due to its intrinsic resistance to acetaldehyde [45]. Equimolar amounts of dimethyl adipate and glycerol were used, in tetrahydrofuran (THF), at 60 °C, with vacuum being progressively applied [50]. The experimental apparatus was equipped with a Soxhlet extractor bearing molecular sieves, which trapped methanol as it formed during the reaction. As previously, at the end of the polymerization, the temperature was increased to deactivate any remaining free enzyme. Except for Novozym 435, Lipozyme^TM^ has also been employed for the polymerization between dimethyl esters and Gly (10% *w*/*w* of diester, 60 °C, reduced pressure) [51]. The advantage of such solvent free protocols is that the reaction could almost be completed in only 7 h. With a similar procedure, other glycols, like 1,2,4-butanetriol and 1,2,6-trihydroxyhexane [44], sorbitol [52] or xylitol [41], were used to prepare aliphatic hydroxyl-substituted polyesters. 

Finally, the third approach involved the polyesterification of glycerol and adipic acid, regioselectively catalyzed by Lipase B (CAL) from *Candida antarctica* [43]. The reaction was initiated by the addition of CAL to an equimolar solution of glycerol and adipic acid in dioxane with molecular sieves (0.4 nm). The reagents, in the form of a suspension, were maintained at 30 °C for 72 h. However, all studied reaction conditions lead to low molecular weight polymers (M_n_ < 1655 g/mol). 

#### 4.1.2. Hyperbranched PGAd

Linear PGAd has attracted a lot of attention and its synthesis and properties have been extensively studied, as described above. However, PGAd can also be prepared as a hyperbranched polymer. In a recent study, Navarro et al. reported the synthesis of hyperbranched PGAd elastomer, using 1:1 and 0.6:1 Gly/AdA molar ratios, at 150 °C, at atmospheric pressure for 17 h and under high vacuum (3 mm Hg) for four more hours (Table 3) [53]. The M_n_ of the obtained PGAd was higher at 1:1 molar ratio while the degree of branching was higher at 0.6:1 molar ratio. In order to prepare elastomeric materials, PGAd prepolymers were crosslinked by heating at 120 °C for 100 h. The extent of crosslinking was followed by weight loss measurements. The prepared elastomers were amorphous and T_g_, mechanical properties and in vitro enzymatic hydrolysis depended on the Gly/AdA molar ratio. PGAd prepared with 1:1 molar ratio had a T_g_ of −15 °C, higher tensile strength and Young modulus and higher mass loss compared with PGAd synthesized with 1:0.6 molar ratio (T_g_ −2.5 °C). Zhang et al. studied the same reaction with [-OH]/[-COOH] stoichiometries varying from 1.5 to 2.2 (corresponding Gly/AdA ratios 1:1–2:1) [54]. The molecular weight and the DB both increased by reducing the [-OH]/[-COOH] ratio. The polymerization was carried out at 140 °C using dibutyltin oxide as catalyst. PGAd hyperbranched polyester is thermally stable since up to 200–300 °C [55]. 

### 4.2. Applications

#### 4.2.1. Linear PGAd and Acylated PGAd Nanoparticles 

PGAd is a biodegradable polyester [56] which exhibits a high biocompatibility. Additionally, it can be degraded by human enzymes, producing non-toxic removable metabolites [57]. For these reasons, it has found many applications as a carrier for drug delivery. When assessed with respect to hemolysis of red blood cells, metabolic activity and growth of human hepatoblastoma cells, it was found that PGAd nanoparticles (NPs) did not show any hemolytic activity up to a concentration of 10 mg/mL [58]. Additionally, all PGAd-based particles tested were nontoxic for HepG2 cells.

To evaluate PGAd nanoparticles as a potential drug delivery system for brain tumor therapy, the cellular uptake, retention, and metabolism of rhodamine B isothiocyanate (RBITC)-labelled NPs were examined in DAOY medulloblastoma cells [59]. RBITC PGAd NPs were prepared by an interfacial deposition method. The cellular uptake of NPs (endosomal/lysosomal compartment) was dependent on the incubation time and the dose of NPs and it was found that the internalization of RBITC-labelled NPs was mostly mediated by an endocytic process. Furthermore, agreeing with results published elsewhere, tumor cells had higher rates of NP uptake than normal host cells [60,61]. After uptake, the release of RBITC from NPs within cells was about 30–40 times faster than the release in cell culture medium, suggesting a rapid breakdown of nanoparticles within the lysosomal compartment of cells. PGAd NPs could be used as localizing therapeutic agents into cells and could provide prolonged drug effects because of their sustained release in physiological conditions and their rapid release when taken up into cells. 

Nanoparticles can be effectively targeted to the liver, and therefore could be used for improving the therapy of hepatic virus infections including hepatitis B and hepatitis C. A fluorescently-labelled linear PGAd polymeric nanoparticle was prepared and used to track NP uptake into human hepatoma cells transfected with replicating hepatitis C virus genomes (Huh7.5), compared with non-transfected cells as a model representing hepatocyte uptake [62]. Confocal microscopy and flow cytometry demonstrated a two-fold increase in uptake of NP in transfected cells compared to non-transfected ones. Therefore, virus transfection enhanced NP uptake into Huh7.5 cells and NP could be considered as a promising delivery system for targeted treatment of hepatitis viruses.

**Figure 3:**
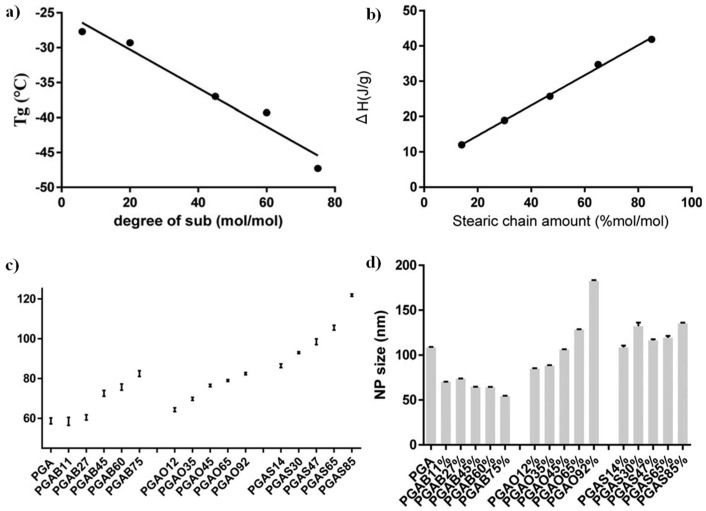
(**a**) T_g_ trends versus butyric residues amount. T_g_ value decreases with degree of functionalization increment. (**b**) First melting peak area (melting enthalpy) versus degree of stearic functionalization. (**c**) Contact angle trend against degree of functionalization and side chain length. (**d**) Particle size of PGA NPs and acylated PGA NPs. Error bars indicate the standard deviation from three measurements. PGAB, PGAO, and PGAS PGA functionalized with butyric (C_4_), octanoic (C_8_) and steraic (C_18_) side chains respectively. Reprinted from [64]. Copyright 2016 The Authors. Journal of Polymer Science Part A: Polymer Chemistry Published by Wiley Periodicals, Inc.

One of the advantages of linear PGAd over PLA and PLGA, which are extensively used in biomedical applications, is the possibility to modify the pendent hydroxyl groups on the polymer backbone with a variety of acyl groups to change the hydrophobicity of the polymer. It is a smart strategy to produce polymers with completely different properties, based on the kind of acyl moieties used [10,42,58,59,62]. The physicochemical properties of the polymer can be adjusted according to the requirements of different drugs (hydrophilic or hydrophobic) allowing various drug incorporations. PGAd itself is hydrophilic but not soluble in water. Variations of the fatty acid chain length and the esterification degree have the potential to provide a wide range of different nanostructured polymeric materials. Due to the nanophase separation between the polymer backbone and side domains, aggregates are formed in water producing a colloidal system. So, these polymers are expected to self-assemble into nanoparticles in the absence of surfactant. Such acylated PGAd copolymers with different chain lengths (C_4_, C_6_, C_8_, C_12_, C_18_, C_22_) have been reported in literature [47,48,50,63,64]. For the acylation, typically linear PGAd was dissolved in THF and the appropriate acid chloride was added (with pyridine to neutralize the eliminated HCl) [64]. The reaction was completed within 3–4 h and, after solvent removal, the prepared acylated derivatives were isolated as viscous oily residues. These were amorphous (C_4_ and C_8_) with a decreasing T_g_ as the degree of substitution increased (Figure 3) or semicrystalline materials (C_18_) with a stable melting point at about 40 °C, that was independent of the degree of substitution. The advantage of these acylated PGAd polymers is that they tend to self-assemble to produce well-defined nanoparticles of high homogeneity, with sizes depending on the degree of substitution and on the length of the alkyl chain. Longer acyl chains or higher substitution degrees should lead to bigger particles due to steric hindrance; however, the resulting stronger hydrophobic interactions could justify a more condensed core and a smaller size. The aggregation or the aggregation number affected the size of the particles as well. It was observed that the diameter of the NPs increased slightly from C_4_ to C_18_. 

**Figure 4:**
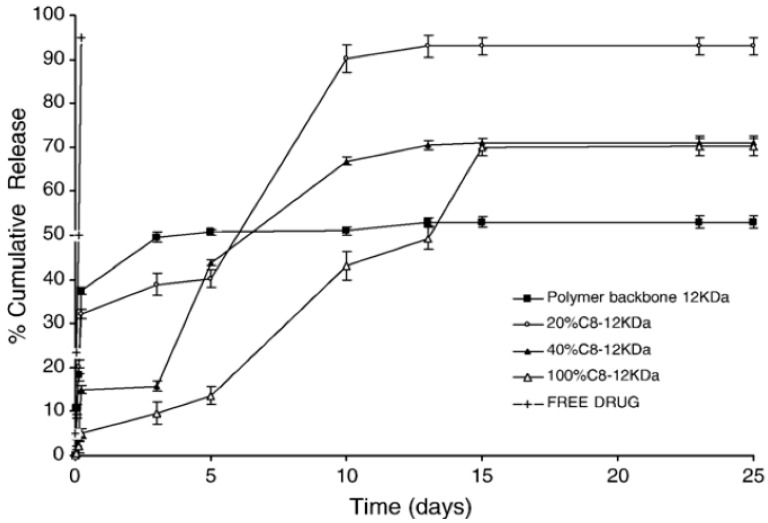
Influence of degree of polymer acylation on dexamethasone phosphate (DXMP) release from nanoparticles. Drug release studies were carried out in water at 37 °C for 25 days. Reprinted with permission from ref. [10]. Copyright 2007 Elsevier Ltd.

The encapsulation of dexamethasone phosphate (DXMP) in PGAd-based polymers for controlled release applications has been investigated both theoretically [65] and experimentally [10,66]. DXMP is the 21-O-phospho derivative of dexamethasone and as such it bears a steroid ring and act as a glucocorticoid receptor agonist; it has thus found many uses in the treatment of cancer. DXMP-containing NPs from acylated and non-acylated PGAd were prepared by the interfacial deposition method [10,66]. Between polymers with different acyl group chain lengths (from C_2_ to C_10_) and varied acylation percentage, the fully acylated PGAd with C_8_ side chains showed the highest drug incorporation (10.5% *w*/*w* drug loading) and the better controlled drug release, due to the strong interactions between the drug and the polymer (Figure 4) [10]. 

Besides DXMP, cytosine arabinoside (Cyt-ara) [10], ibuprofen, its sodium salt (IBU-Na), and ketoprofen [67] were encapsulated in both unmodified and substituted PGAd polymers, by the interfacial deposition method. When it comes to Cyt-ara, the highest molecular weight polymer was the most suitable; unlike DXMP, acylation had a negative effect on the performances of the polymer, and NP from unmodified PGAd exhibited the higher incorporation and the best release profile. For ibuprofen, IBU-Na, and ketoprofen clear trends were not observed [67]. IBU-Na was molecularly dispersed inside the nanoparticles, which was attributed to the strong interactions that took place with the polymer reactive groups, while the other two drugs were dispersed in crystalline state. The drug release from PGAd and acylated PGAd derivatives differed and showed stepwise patterns that involved an initial burst release, diffusion of the drug from the polymer matrix and eventually drug release possibly via a combined mechanism. Acylated PGAd NPs were also used in antiviral therapy, for the delivery of ribavirin, a broad antiviral agent active against both RNA and DNA viruses [42]. 

#### 4.2.2. PGAd Conjugates 

**Scheme 6:**
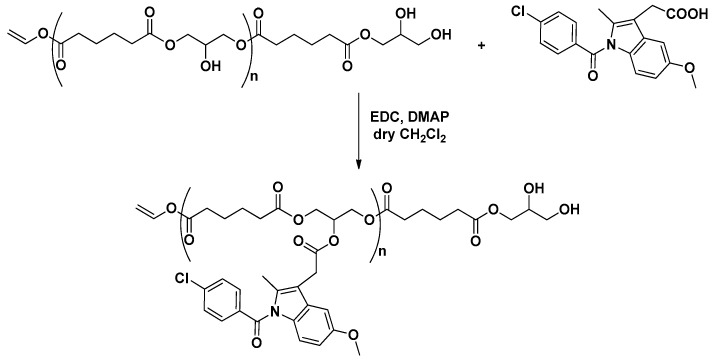
Conjugation of indomethacin to PGAd [68]. (EDC: *N*-(3-dimethylaminopropyl)-*N*′-ethylcarbodiimide, DMAP: 4-dimethylaminopyridine).

The free secondary hydroxyl groups in linear PGAd can be also used to prepare drug-polymer conjugates. In this case, the drug is released via the hydrolysis of a covalent bond and its release can be extended and better controlled. Wersig et al. presented a comprehensive investigation of the properties of PGAd–indomethacin conjugates in comparison to the drug free backbone polymer [68,69]. PGAd secondary hydroxyl groups were esterified to different degrees (25%, 50% and 100%) with the anti-inflammatory drug indomethacin (Ind), Scheme 6, in order to create a prodrug with a pH-sensitive linker for modified drug release. The catalyzed esterification afforded a good control over the drug loading and allowed the regulation of the properties of the polymer for further processing. All conjugates were amorphous with a T_g_ that increased with increasing Ind content. Drug release was monitored over one month in vitro. A very low release rate was observed in phosphate buffer saline (PBS). Slightly acidic conditions and lipase presence increased Ind release. Therefore, PGAd–Ind conjugates are promising prodrugs for the local sustained release of indomethacin.

These PGAd-Ind conjugates were further used to prepare nanospheres for the parenteral delivery of Ind [69]. The results indicated that PGAd-Ind conjugates were successfully formulated as nanospheres with a small particle size distribution in the range of 200 nm, suitable for intravenous application. The produced nanospheres had a high drug loading and performed well in biological in vitro tests (hemolysis and cell toxicity in three cell lines). A controlled drug release without any burst was observed in vitro over 15 days in phosphate buffer pH 7.4. The newly developed particles have the potential to improve the therapy of inflammation and associated diseases. 

Suksiriworapong et al. reported a PGAd-anticancer drug conjugate with methotrexate (MTX) with MTX% amount up to 27.5 mol %, which was prepared by a simple carbodiimide coupling reaction in DMSO [57]. It was found that MTX-PGAd conjugate self-assembled into nanoparticles whose size depended on the amount of conjugated MTX and the pH of medium. In vitro experiments demonstrated that MTX-PGAd nanoparticles showed seven times higher toxicity to Saos-2 cell lines than free MTX.

Besides linear PGAd, hyperbranched PGAd was recently used as a matrix for the sustained release of 2-undecanone and salicylic acid, which were covalently bonded to the hydroxyl end groups of PGAd [70]. The release of both molecules took place by chemical or enzymatic hydrolysis while their release rate depended on the composite structure and the amount of catalyst used.

#### 4.2.3. PGAd Copolymers

PGAd can be polymerized with other monomers to prepare advanced copolymers. For example, in a recent study, poly(glycerol adipate-co-*ω*-pentadecalactone) (PGAd-co-PDL) copolymers were used for bovine serum albumin (BSA) nanoencapsulation within l-leucine (l-leu) microcarriers for dry powder inhalation [71]. NPs of size 128.50 ± 6.57 nm, suitable for targeting lung dendritic cells, were prepared by oil-in-water single emulsion solvent evaporation. More than 90% of BSA was released within 48 h due to weak hydrophobic interactions between BSA and the NPs while the released BSA retained approximately 77% of relative residual esterolytic activity, compared to standard BSA. The results suggested that PGAd-co-PDL/l-leu system may be a promising carrier for pulmonary vaccine delivery due to the excellent BSA adsorption and aerosolization behavior. Indomethacin was also microencapsulated in a similar copolymer [72]. 

The covalent and noncovalent coating of stearic acid-modified PGAd (PGAdS) nanoparticles with N-(2-hydroxypropyl) methacrylamide (HPMA) copolymers was recently reported [73]. This work aimed to investigate the fate of PGAd NPs in living organisms and how coating could affect it. The size of the particles was not much affected by the HPMA coating, but the zeta-potential values significantly decreased from −60 to −10 mV and the pharmacokinetics and biodistribution of the particles were also influenced. Unlike coated NPs, uncoated PGAdS NPs showed visible accumulation in the bones at early time points. In vitro studies indicated an interaction with calcium ions, which could be blocked with hydroxyapatite. The desired tumor accumulation was observed for all NPs, but accumulation in the ovaries, adrenal glands, and liver was also observed. 

## 5. Poly(Glycerol Suberate), PGSub 

Poly(glycerol suberate) has not been as extensively studied as other glycerol polyesters, such as poly(glycerol adipate) or poly(glycerol sebacate). To the best of our knowledge, the only report of PGSub is a recent paper of Unnisa et al. [74]. PGSub was synthesized by the reaction of glycerol with suberic acid in 1:1 ratio at 140 °C under nitrogen flow for several hours in order to get a homogeneous solution. The polyester was characterized by several techniques and was used to prepare blends with poly(vinyl alcohol) (PVA) and doped with various amounts of NH_4_SCN, to prepare an electrolyte for proton conducting batteries. A maximum conductivity of 3.01 × 10^−4^ S/cm was reported for 1 g PVA + 0.75 g PGSub + 0.6 g NH_4_SCN.

## 6. Poly(glycerol Azelate), PGAz 

Azelaic acid (AzA) can be made from renewable resources (ozonolysis of oleic acid, the most common of the unsaturated fatty acids) and is suitable for biomedical applications because of its antibacterial properties. Wyatt and his research group reported the polycondensation of glycerol with azelaic and succinic acids in order to prepare hyperbranched oligomers [19]. As for succinic acid (vide supra), azelaic acid was reacted with glycerol, in a 1:1 molar ratio, in the presence of titanium(IV) butoxide (0.15% *w*/*w*), starting at 60 °C and progressively heating up to 150 °C, under reduced pressure (total reaction time 16 h) (Table 4). The prepared Gly/AzA oligomers (M_n_ = 2316) were not water-soluble but were soluble in nonpolar solvents such as toluene. Seeking to increase the degree of branching of PGAz oligomers but without increasing crosslinking, the polymerization reaction was performed in aprotic polar solvents (DMSO or DMF) and higher M_n_ were obtained (Table 4) [33]. In the same study it was reported that PGAz oligomers had a slightly higher DP when compared to the oligomers synthesized from succinic and glutaric acids. The effect of a non-polar solvent (toluene) on the condensation of azelaic acid with glycerol was studied as well [18,35]. GPC provided evidence that increasing molar ratios of Gly/AzA and reaction time, increased the molecular weight and degree of polymerization and decreased the residual glycerol. The 1:2 Gly/AzA formulations gave the products with the highest D.B.: 13.9% at 135 °C for 24 h and 48.5% for 72 h [35]. Unlike what was observed in DMF, when compared to succinic and glutaric acid, it was demonstrated that the DP increased with decreasing aliphatic chain length and that the polymerizations involving azelaic acid were the slowest. 

Kadhum et al. studied a catalyst-free polycondensation of azelaic acid with glycerol and how this affected the thermal stability [76]. The thermal stabilities of all three samples were comparable (>385 °C), with the sample synthesized with a 1:1 Gly/AzA molar ratio exhibiting the highest one (408 °C). Chongcharoenchaikul et al. evaluated the effect of the Gly/AzA molar ratio and the curing duration of a catalyst-free polycondensation reaction on the chemical structure, mechanical properties and biodegradability of the resulting PGAz sheets [75]. The highest M_w_ was measured for the prepolymers prepared with a 1.5:1.0 molar ratio and a reaction time of 16 h (8527 g/mol). The T_g_ value was measured by dynamic mechanical analysis (about −24 °C) and was shifted to higher temperatures as the Gly/AzA molar ratio increased from 1:1 to 1.5:1, which is due to the increased rigidity of the dendritic units in the molecular chains. Young’s modulus and tensile strength were increased by increasing the curing time from 24 h to 48 h (0.98 to 4.14 and 0.26 to 0.85 MPa, respectively) as well as by increasing Gly/AzA molar ratio (from 3.62 to 11.11 and 0.44 to 0.66 MPa, respectively) while elongation at break followed the opposite trend [18]. The Young’s modulus of the PGAz sheet was higher than that reported for poly(glycerol sebacate) (PGS) and the degradation rate slightly lower. A Young’s modulus in the range of 2–46 MPa is suitable for applications as collagen fiber scaffolds in tendons, cartilage, ligaments and bones. Thus, the increased stiffness of PGAz makes it a suitable candidate for collagen fiber scaffolds. The rapid degradation of PGAz may be an advantage in drug delivery applications, where the release of the encapsulated drug occurs by degradation of the carrier vehicle. Additional applications of PGAz polymers are expected in cosmetics, food additives, surfactants, lubricants, etc.

## 7. Poly(Glycerol Sebacate), PGSeb

Poly(glycerol sebacate), PGSeb, was thrown in the limelight in 2002 by the Langer lab, at the Massachusetts Institute of Technology [77], it has attracted considerable interest since and it is the most studied hyperbranched polyester till today [5,78,79,80,81]. Sebacic acid (SeA), HOOC(CH_2_)_8_COOH, is a metabolite in *ω*-oxidation of medium- to long-chain fatty acids and as a result, PGSeb is biocompatible. The FDA has approved polymers containing sebacic acid for medical applications.

### 7.1. Synthesis and Properties 

#### 7.1.1. Synthesis 

Wang and his coworkers reported that the polycondensation of glycerol and sebacic acid in a 1:1 molar ratio afforded a transparent, almost colorless, elastomer [77]. The polycondensation was carried out at 120 °C, at atmospheric pressure for the first 24 h to yield a prepolymer, which was subsequently cured, at 120 °C, under vacuum for another 48 h. During the curing step, crosslinks between linear chains are created due to the esterification of the secondary hydroxyl group. Additionally, transesterification reactions take also place, as confirmed by the collection of glycerol with the water removed by vacuum [82,83]. After curing, the resulting polymer had a crosslinked three-dimensional network in combination with random coil characteristics. The crosslinking density was low, and a lot of hydroxyl groups, mainly secondary OH, were left unreacted, rendering the polymer rather hydrophilic. FTIR analysis showed that these OH groups are hydrogen-bonded (broad peak at 3448 cm^−1^). PGSeb achieved a Young’s modulus, an ultimate tensile stress (UTS), and a rupture strain of 0.282 MPa, >0.5 MPa, and >267%, respectively. Differential scanning calorimetry (DSC) indicated that PGSeb is soft and amorphous at 37 °C and it was characterized as a thermoset elastomer.

Different kind of glyceride ester units can be found in the PGSeb prepolymer and polymer. ^13^C NMR was used to determine the ratio of 1-substituted, 1,2-disubstituted, 1,3-disubstituted, and trisubstituted glycerol units in the PGSeb polymers [84,85] and to observe how it changed as the polycondensation progressed [83]. As expected, as the reaction proceeded (prepolymer vs. cured polymer) the percentage of glycerol and monoacylglycerides decreased while the diacyl and triacyl units increased. In the prepolymer, the 2-monoacylglyceride unit (~3%) was present in lower concentrations than the 1-monoacylglyceride confirming the higher reactivity of the primary hydroxyl groups towards esterification. After curing, the 1,3-diacylglyceride unit had the highest concentration. The concentration of the 1,2,3-triglyceride unit was higher in the cured polymer than in the prepolymer.

Kafouris et al. studied PGSeb prepolymers by mass spectroscopy (MS) [6]. Low molecular weight oligomers were detected, mainly with 2–8 repeating units and no cyclic structures. Later, Moorhoff et al. used high resolution MS (positive and negative mode ion electrospray ionization and atmospheric pressure chemical ionization) to analyze PGSeb prepolymers [86]. The tendencies observed by Kafouris et al. [6] were confirmed, though the results were different, probably due to the different synthesis protocols.

**Figure 5:**
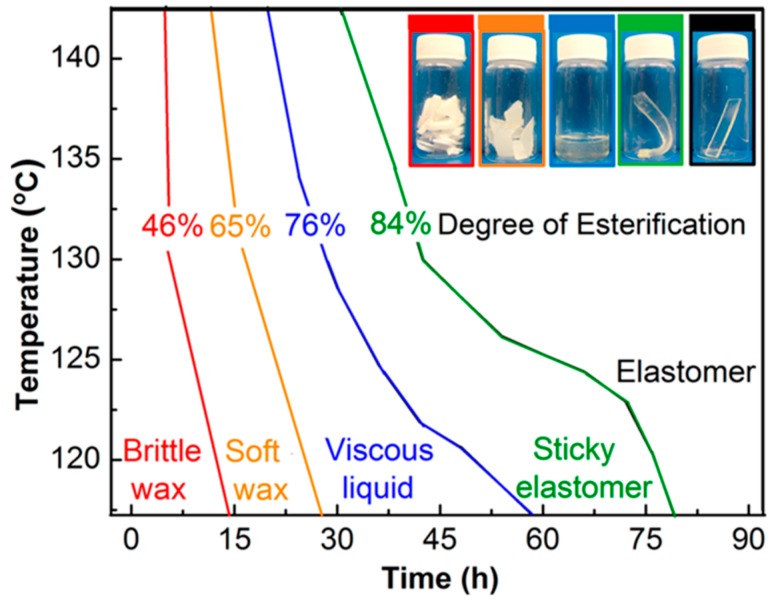
Map describing the relationship between the esterification degree and the specimen’s status. Three temperatures (prepolymerization and curing) were studied: 120, 130, and 140 °C. Reprinted with permission from [94]. Copyright 2015 American Chemical Society.

Determining the extent of the reaction or the degree of esterification (degree of branching) is important as it is related to the crosslinking density or strand density, which is closely linked to the mechanical properties of the polymers (vide infra). Nagata et al. used the FTIR ratio between the -OH peak and the -CH_2_/CH peak to estimate the degree of reaction [87,88]. Maliger et al., also employing IR, calculated the extent of the esterification reaction based on the peak corresponding to the ester moiety [89]. Liu et al. used ^1^H NMR to demonstrate that in the prepolymer residual hydroxyl groups were present (esterification degree 54% for primary OH and 11% for secondary OH) [90,91]. Kafouris et al. determined the conversion of the reaction based on ^1^H NMR [6], Maliger et al. used MALDI-TOF spectroscopy [92], while others titrated unreacted -COOH groups to determine the extent of esterification [83,93,94]. Monitoring the mass of water that condenses during the preparation of the polymer, and comparing it to the amount that should be produced is another method used to calculate the degree/extent of esterification [83,95]. Li et al. prepared PGSeb samples with a range of esterification degrees and mapped the physical state of the resulting polymers (Figure 5) [94]. This “map” was conceived as a guide in order to choose a thermal treatment and esterification degree, depending on the desired physical state of PGSeb. The observed differences were accompanied by solubility differences and were related to the increasing chain length and extent of crosslinking. More recently, Gadomska–Gajadhur et al. developed mathematical models to calculate the expected esterification degree based on the reaction temperature, the reaction time and the molar ratio of monomers and the conversion of carboxylic acid groups depending on the reaction temperature and monomer ratio [96]. 

Maliger et al. studied the kinetics of the esterification of glycerol with sebacic acid in a toluene solution, for different Gly/SeA ratios and temperatures and observed two regimes [89]. The kinetically controlled regime, where first order kinetics were observed, and the diffusion-controlled regime, where the viscosity had significantly increased and mass transfer limited collisions of the reactive species. When the temperature increased, the rate increased and the conversion during the kinetically controlled regime increased. When the Gly/SeA ratio increased, the average functionality (calculated according to the Pinner equation) decreased and hence the crosslink density decreased as well, resulting in lowering of the activation energies. 

The influence of the molar ratio of glycerol and sebacic acid on the molecular weight or thermal properties of the resulting PGSeb has not been studied as much as with PGAd or PGSuc (vide supra). Most authors have worked with a 1:1 molar ratio, optimizing other reaction parameters, such as temperature and curing time. When it comes to enzymatic catalysis, Uyama et al. observed that by decreasing the Gly/SeA molar ratio from 1 to 1:1.5, the M_w_ decreased (approximately from 19,000 to 4500) [85]. Maliger et al. observed that the M_w_, derived from Carother’s equation, increased when the Gly/SeA increased from 0.6 to 0.8 but a further increase to 1.0 led to a decrease (polymerization in toluene solution) [89]. A similar trend was also observed by Kafouris et al. who studied the molecular weight of prepolymers for Gly/SeA ratios ranging between 2:1 and 2:5 [6]. A decrease in T_g_ was reported by Liu et al. [97] for Gly/SeA ratios decreasing from 1:1.25 to 1:2 (Table 5), which was further confirmed by Conejero–García et al. [98]. 

The synthesis of PGSeb necessitates long reaction times at high temperatures and it is a time-consuming and energy-demanding process. Some authors have explored the microwave assisted synthesis of PGSeb [94,95,99]. The use of microwave irradiation significantly increased the reaction rate, producing polymers with a higher esterification degree in less time. However, important glycerol evaporation, resulting in a distortion of the molar ratio of glycerol and sebacic acid in the final polymer, was observed. What is more, Lau et al. demonstrated that microwave irradiation promoted a higher DB compared to conventional heating [95]. Indeed, owing to a comparable polarity, primary and secondary hydroxyl groups of glycerol were activated in a similar degree by microwave irradiation, resulting in an equivalent reactivity. The authors concluded that microwave irradiation, besides offering shorter reaction times, also allowed for a higher flexibility in the tuning of the mechanical properties and degradation rates of PGSeb. Microwave irradiation could considerably reduce curing time as well [100]. 

When the polymerization of glycerol and sebacic acid was carried out in a BrabenderPlasticoder^®^, due to high-shear mixing, an increase in the rate constant and degree of polymerization was noted [92]. Finally, Uyama et al. studied the lipase-catalyzed reaction of divinyl sebacate with glycerol, which afforded polymers with average molecular weights ranging from 1100 to 19,000 depending on the temperature (45 or 60 °C) and the origin of the lipase used [84,85]. 

#### 7.1.2. Mechanical Properties

Several groups have studied how the molar ratio of glycerol and sebacic acid, the synthesis and curing temperatures and the curing duration may affect the properties of the resulting polymer. The mechanical properties are determined by the combined effect of the sol content (soluble part), the crystallinity, and the crosslinking degree. An important sol content deteriorates the mechanical properties, but it is necessary for the thermal moldability of a crosslinked polymer [91].

According to Liu et al., when the molar ratio Gly/SeA decreased, more hydroxyl groups were esterified by sebacic acid [97]. This had two competing effects: i) more crosslinking points were formed; ii) more soluble oligomers with a certain molecular weight were produced (because when the hydroxyl groups were esterified, they could not further react). As a result, the elastic modulus, E, increased with decreasing molar ratio and the elongation at break, ε, decreased. The tensile strength, δ, increased until the molar ratio of 2:3.5 and decreased afterwards (Table 5).

These observations are consistent with the results of Maliger et al. and Kafouris et al. Kafouris and his coworkers showed that when the molar ratio of -COOH to -OH groups was the closest to 1 (Gly/SeA molar ratio: 2:3) the stiffest material (highest storage modulus) was obtained due to a high crosslink density [6]. Maliger et al. provided evidence that the Young’s modulus increased as the molar ratio Gly/SeA decreased and the average functionality increased, (Table 6) [89]. It was further shown that adding sebacic acid during the curing step could improve the mechanical properties (Table 5) [90,91]. 

Except for the molar ratio of the monomers, polymerization duration and temperature, and curing also affected the properties of PGSeb to a high extent. Varying the polymerization duration yielded prepolymers with different molecular weights, resulting in reaction mixtures differing in viscosity and reactivity, affording polymers with different mechanical properties [91]. Synthesis at higher temperatures (130 °C) yielded rigid, highly crosslinked polymers while lower temperature (110 °C) afforded PGSeb exhibiting characteristics of soft compliant elastomers (Table 6) [101]. Interestingly, it was shown that PGSeb samples with the same strand density, but pre-polymerized at a lower temperature (130 vs. 150 °C) were characterized by higher values of ultimate tensile strength [83]. This was attributed to a more important loss of glycerol when the synthesis took place at a higher temperature, which in turn resulted in a more important concentration of longer network strands that increased the strength of the sample. 

When it comes to curing, increased curing duration resulted in more highly crosslinked polymers [102], with increased equilibrium Young’s modulus [91], tangent modulus [103] and average ultimate tensile stress (UTS) [83,104] and a diminished strain at break (*ε*_max_) [104]. The general idea is that increased curing time is synonym to increased stiffness [98,105,106,107]. However, increasing curing time or temperature did not improve PGSeb properties indefinitely [98]. Chen et al. demonstrated that, with increasing curing time, the number of crosslinks, measured by the strand density, increased rapidly, before reaching a saturated plateau [83,104]. 

Kemppainen et al. found that the tangent modulus (at 10% strain) could be predicted according to equation 1 [103], while Jaafar et al. quantified the relationship between the IR maximum peak height of the hydroxyl stretch and the respective equilibrium modulus [102]. Mitsak et al. demonstrated that the behavior of PGSeb could be modeled using a non-linear elastic Neo–Hookean model [106]. Finally, Li et al. showed that there is a linear relationship between the elastic modulus and the degree of esterification [94].

(1) y=3.607 − 1.410 × (molar ratio Gly:Seb)+0.60 × (curing time in hours)

Wet PGSeb samples exhibited lower mechanical properties than dry ones [106]. Increasing porosity resulted in a decrease of the elastic properties of PGSeb [106]. Besides the mechanical properties, a decreasing amount of sebacic acid increased the hydrophilicity of PGSeb (this can also be observed in Table 5), thus favoring cell growth and adhesion [108]. 

In conclusion, the mechanical properties depend significantly on the crosslink density which is adjustable through the synthesis and curing conditions. Therefore, the mechanical properties of PGSeb, depending on the targeted application, can be tailored during the synthesis of the polymer. Finally, it was shown that the properties of PGSeb are not affected by common sterilization methods [109].

#### 7.1.3. Degradation

In tissue engineering applications, the degradation rate and mechanism of the scaffold is critical. Ideally, the degradation kinetics of the artificial scaffold should match the healing rates of the injured tissue. PGSeb degraded slowly in vitro but faster in vivo: agitation for 60 days in PBS solution at 37 °C caused the polymer to degrade by 17% (as measured by change of dry sample weight) [77] but, in in vivo studies, PGSeb implants were totally absorbed within 6–8 weeks [110,111]. This difference is attributted to the presence of enzymes (esterases and oxidoreductases) that catalyzed the hydrolysis of ester bonds in PGSeb. Crosslinking density controlled significantly the degradation rate, with polymers having a higher crosslink density exhibiting a slower degradation [101,107,112]. 

The degradation of PGSeb is dominated by surface erosion [113]. Porous PGSeb samples were reported to lose their surface porosity (six weeks) before their inner porous structure (10 weeks) [114]. The swelling of PGSeb indicated some degree of hydrolysis inside the polyester network (bulk degradation). However, this process is slow compared to the enzyme catalyzed surface degradation. In other words, fast enzyme catalyzed surface erosion took place in parallel with slower bulk degradation. It is notable that the mechanical properties did not deteriorate linearly with mass loss [113,115]. 

To sum up, PGSeb degrades via surface erosion, retaining its mechanical properties. The degradation kinetics of PGSeb could be tailored through the polycondensation processing (curing conditions and/or reactants ratio) to satisfy the requirements of each specific application.

#### 7.1.4. Compatibility

All compatibility studies have put forward an excellent biocompatibility, both in vitro and in vivo [77,116]. In in vitro studies, cells were found to proliferate at satisfactory rates on PGSeb [117]. In in vivo studies, no gross inflammation was observed and, unlike PLGA, which is widely used in biomedical research, PGSeb induces minimal fibrosis around the implant [110]. 

Some authors have however reported a slight toxicity, which was attributed to a local pH decrease [118,119,120]. This pH decrease could be due to unreacted COOH groups in the polymer, to the release of sebacic acid, originating from unreacted monomer, to PGSeb degradation [107], or to uncrosslinked oligomers. Pre-conditioning the PGSeb samples was suggested to override this inconvenience [118,121]. Li et al. reported that increasing curing time improved the cytocompatibility of PGSeb samples [83]. 

#### 7.1.5. Processing 

One of the advantages of PGSeb is the ease of processing. PGSeb prepolymer can be melted or dissolved for further fabrication processes. The most widely used techniques are replica molding and laser ablation, however a variety of different processes have been used to shape PGSeb.

**Figure 6:**
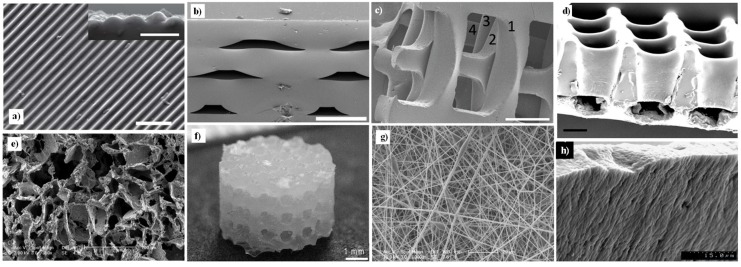
SEM and CT images of PGSeb scaffolds. (**a**) Replica-molded substrate. The sharp features in the silicon mold become rounded due to the effect of the sucrose release layer. Inset show feature heights and periods. Reprinted from [122]. Copyright 2005, with permission from Elsevier. (**b**) Cross-sections of three-layer device. Reprinted with permission from [128]. Copyright 2006, WILEY-VCH Verlag GmbH & Co. KGaA, Weinheim. (**c**) Four-layered scaffolds, with numbers indicating individual layers. Reprinted with permission from [129]. Copyright 2013, WILEY-VCH Verlag GmbH & Co. KGaA, Weinheim. (**d**) Bilaminar scaffold with 3D pore networks fabricated through a combination of excimer laser microablation and lamination, with fully interconnected 200 × 200 µm top-to-bottom and 200 × 100 µm lateral pores [126]. Reprinted with permission from Springer Nature, copyright 2008. (**e**) Scaffold obtained by the salt leaching procedure, exhibiting extensive interconnected macropores of 75–150 μm in diameter. Reprinted with permission from [131]. Copyright 2007, Wiley periodicals Inc. (**f**) Micro-CT image of 3D scaffolds obtained by a wax mold. Reprinted with permission from [103]. Copyright 2010, WILEY-VCH Verlag GmbH & Co. KGaA, Weinheim. (**g**) PGSeb-poly(L-lactide)(PLLA)/PLLA core/shell nanofibers. Reprinted with permission from [132]. Copyright 2008, WILEY-VCH Verlag GmbH & Co. KGaA, Weinheim. (**h**) Anisotropic membrane obtained in the presence of aligned poly(vinyl alcohol) (PVA) fibers. Reprinted with permission from [133]. Copyright 2017, John Wiley and Sons. Scale bars: (**a**) 10 μm in the large figure and 5 mm in the inset, (**b** and **e**) 200 μm, (**c** and **d**) 100 µm, (**f**) 1 mm, (**g**) 20 µm.

Several groups have used a silicon-based replica molding process to prepare PGSeb scaffolds: PGSeb prepolymer, molten or in solution, was cast on a silicon master with the appropriate features, the prepolymer was cured, and the final object delaminated from the silicon master (Figure 6a). The use of a sacrificial layer (sucrose or maltose) between the silicon wafer and the prepolymer has proved to be beneficial when releasing the polymer from the wafer [105,122,123,124]. 

PGSeb is a thermally stable polymeric material and is well adapted to laser microablation, affording scaffolds with high edge quality and smooth surfaces [125]. For example, Engelmayr et al. patterned pores on PGSeb membranes used laser microablation to create an accordion-like honeycomb scaffold [126,127]. 

Three-dimensional scaffolds can be constructed by stacking and bonding individually prepared 2D scaffolds. For example, Bettinger et al. combined micropatterned PGSeb sheets to form a three-dimensional capillary network (Figure 6b) [124,128]. Kolewe et al. reported a semi-automated method to assemble porous PGSeb sheets to form multi-layered scaffolds with interconnected pores, (Figure 6c) [129]. The PGSeb layers can be bonded together through an additional thermal curing step [128,130], by oxygen plasma-mediated lamination [127] or solvent bonding (dip-coating a PGSeb patterned layer into a solution of prepolymer and then bringing it into contact with a PGSeb membrane, followed by thermal and vacuum curing) [121]. Oxygen plasma-mediated lamination uses lower temperatures (room temperature), allowing for PGSeb scaffolds with a lower stiffness. Nevertheless, Ye et al. supported the superiority of the solvent bonding approach based on SEM imaging, flow tests and mechanical properties [121]. Bilaminar scaffolds with 3D pore networks were also fabricated by laser microablation. A lateral array of troughs was microablated in one PGSeb lamina, a second PGSeb lamina was overlaid over the first one and pores were microablated top-to-bottom through both laminas (Figure 6d). The resulting scaffold was stabilized by thermal crosslinking [126,127]. 

Macroporous PGSeb scaffolds with extensive micropores were prepared by curing PGSeb dispersed throughout a fused salt particle template (Figure 6e) [82,131,134,135]. Salt particles, used as porogens, were introduced in a mold and fused. A tetrahydrofuran PGSeb prepolymer solution was added to the fused salt templates. After the evaporation of THF, PGSeb is cured by heating under vacuum Finally, PGSeb-impregnated salt templates were soaked in water to remove the salt particles. Porosity can be controlled through the mass ratio of salt particles and besides increasing porosity, a higher mass ratio results in increased pore interconnectivity [136]. SEM photographs of scaffolds prepared by salt leaching showed the presence of many micropores distributed throughout the walls between the macropores. The micropores were most likely created by the removal of glycerol generated by transesterification during the curing step [131]. 

Hollister and his coworkers used wax molds to obtain a 3D porous scaffold [103,137]. Wax molds of the final scaffold design were made and pressed into hydroxyapatite (HA), resulting in an inverse HA mold. The inverse HA mold was pressed into PGSeb prepolymer and the PGSeb prepolymer/HA mold unit was placed within a vacuum oven to cure the prepolymer. Finally, the HA was dissolved using a decalcifying agent to afford the final PGSeb scaffold (Figure 6f). 

Frydrych and Chen applied a freeze-drying technique to elaborate porous three-dimensional scaffolds [138]. Briefly, PGSeb prepolymer and PLA were dissolved, the solution was freeze-dried, and the obtained scaffolds were cured in a vacuum oven at 150 °C. The PGSeb/PLA scaffolds obtained were highly porous with interconnected pores and exhibited soft elastomeric properties. However, upon removal of PLA, the integrity of the scaffolds was compromised, and they eventually collapsed.

**Figure 7:**
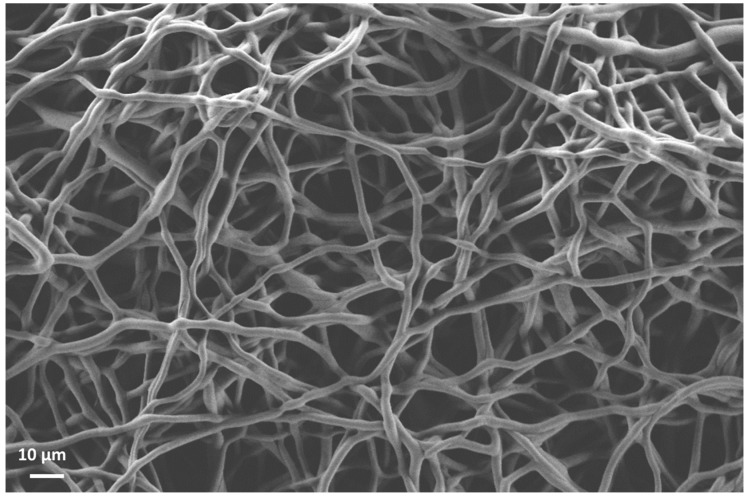
SEM micrograph of electrospun PCL/PGSeb fibers by using acetic acid as solvent, obtained by the authors following the protocol reported by Vogt et al. [143]. Data not previously published.

Several authors explored the possibility to prepare PGSeb fibrous scaffolds in order to mimic the extracellular matrix (ECM) and contribute to cell alignment in grafts for tissue engineering. Electrospinning cannot be directly applied to crosslinked PGSeb because it is insoluble in any organic solvent suitable for the electrospinning process. On the other hand, PGSeb prepolymer has a low molecular weight, giving rise to low viscosity solutions and “short” polymer chains that do not entangle appropriately. Yi and LaVan reported a coaxial electrospinning process, to prepare PGSeb fibers using PLLA as a protective shell which was removed after the PGSeb prepolymer core was cured (Figure 6g) [132]. Alternatively, PLA-poly(ethylene oxide) (PEO) [139] and PVA have been used [140,141]. PGSeb fibrous sheets were also obtained by electrospinning a PVA/PGSeb prepolymer solution in 1,1,1,3,3,3-hexafluoroisopropanol [142]. Electrospinning of PGSeb blends with other polymers has been extensively studied, but is out of the scope of the present review. However, among those efforts it is noteworthy to mention studies on the use of benign solvents for the electrospinning of blends containing PGSeb prepolymer and mildly crosslinked prepolymer. Vogt et al. reported acetic acid as a solvent for the fabrication of PCL/PGSeb electrospun fibers suitable for soft tissue engineering applications, as cardiac tissue engineering [143]. The microstructure of the electrospun fibers obtained from the blend of PCL and PGSeb in acetic acid is shown in Figure 7.

Using PVA nanofibers as a template, Hsu et al. reported the fabrication of an anisotropic PGSeb membrane: a sacrificial layer of aligned PVA fibers was immersed in the PGSeb prepolymer and, after curing, removed by water, leaving aligned cylindrical pores in the PGSeb membrane (Figure 6h) [133]. The anisotropic PGSeb membranes exhibited a different Young’s modulus and elongation at break along the pore direction versus perpendicular to the pore direction, but the ultimate tensile strength was the same in both directions.

Louage et al. report the successful preparation of PGSeb nanoparticles, ranging from 100 to 200 nm, by diluting an ethanolic solution of PGSeb in water [144]. The nanoparticles were found to be stable in aqueous medium for prolonged periods of time.

### 7.2. Applications

Many soft tissues in the body have elastomeric properties and therefore PGSeb, which is a compliant, biocompatible and bioresorbable material, with a tunable degradation, is a material of choice in the context of soft tissue regeneration. Additionally, since it is a biodegradable polymer, applications for drug delivery were investigated.

#### 7.2.1. Tissue Engineering

##### Myocardial Tissue Engineering

Any biomaterial to engineer the myocardial tissue, which beats cyclically and constantly, should possess long-term elasticity. Since PGSeb could support the activity of human embryonic stem cells-derived cardiomyocytes [118,145] and could be tuned to the appropriate stiffness range (0.04 to 1.2 MPa) [101], several research groups developed PGSeb scaffolds for application as a heart patch material. 

**Figure 8:**
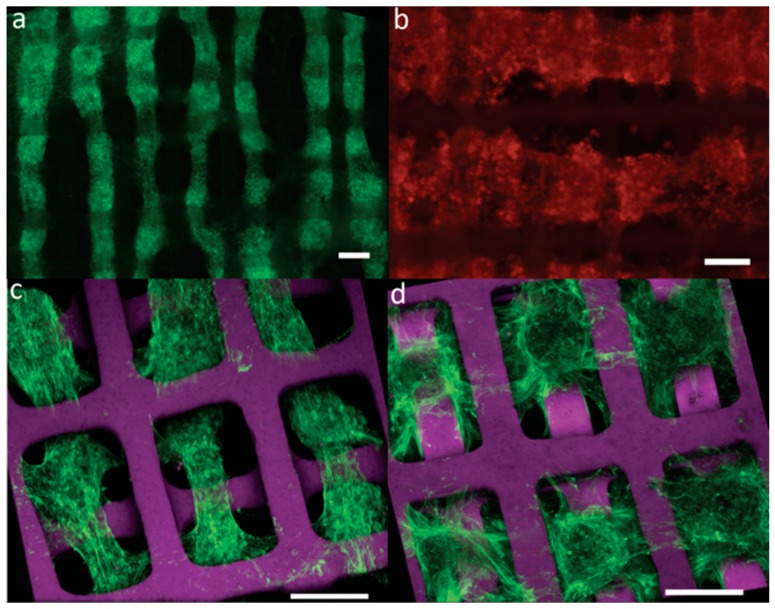
Alignment of neonatal rat heart cells cultured on two-layered PGSeb scaffolds imaged by: (**a**,**b**) fluorescence or (**c,d**) confocal microscopy of: (**a**,**c**,**d**) F-actin stained (green) and nuclear DNA counterstained or (**b**) sarcomeric α-actinin stained (red). Scale bars: 100 μm [129]. Reprinted with permission from WILEY-VCH Verlag GmbH & Co. KGaA, Weinheim. Copyright 2013.

It was demonstrated that scaffolds of low stiffness PGSeb allowed the greatest amplitude of contraction [146]. Besides the stiffness of the scaffold, all the work in the field underline that the scaffold pattern and architecture is of paramount importance as it affects significantly the growth and alignment of cells (Figure 8) [129,147,148]. Stuckey et al. employed magnetic resonance imaging (MRI) to monitor the in vivo performance of PGSeb patches grafted on the hearts of infarcted rats and demonstrated that PGSeb is efficient in preventing post-infarction hypertrophy [111]. As ventricular myocardium exhibits directionally dependent electrical and mechanical properties, several research groups sought to build scaffolds with anisotropic structural and mechanical properties. Engelmayr et al. explored an accordion-like honeycomb pattern [126] while Neal et al. studied two-layers constructs, with anisotropic, interconnected, cubic rectangular, or hexagonal rectangular pores [124]. Ravichandran and his coworkers investigated the use of PGSeb nanofibers [149], while Boccaccini et al. were interested in electrospun fibrous scaffolds [143,150,151]. PGSeb fibers could be directly injected into the infarct wall, avoiding the need of more invading surgical procedures and limiting cell loss. Recognizing the importance of vascularization, Radisic et al. reported a scaffold with a channel array to mimic the role of capillary network [131]. 

##### Vascular Tissue Engineering

Endothelial cells and smooth muscle cells adhere to and proliferate on PGSeb films and scaffolds, reaching confluence, promoting the synthesis of extracellular matrix and exhibiting the expected morphologic and phenotypic properties, demonstrating the potential of PGSeb for vascular tissue engineering [117,130]. Tubular porous scaffolds were successfully fabricated for such applications [134]. It was demonstrated that both the porosity [152] and the compliance [135] of the scaffold affected cell culture. Additionally, evidence was provided that short-term culture of smooth muscle cells could increase the PGSeb scaffold stiffness [135] and that culturing smooth muscle cells on small-diameter PGSeb scaffolds at increased hydrostatic pressure, increased the burst pressure and the compliance of the scaffold [153]. 

Ye and his coworkers engineered a PGSeb scaffold with an integrated vasculature. The prepared scaffold borne a distinct parenchymal compartment and microvessels, separated by a PGSeb semi-permeable membrane. In vitro experiments demonstrated that the microvessels support the cultivation and differentiation of cells in the parenchymal compartment [121]. 

Finally, Wu et al. reported a cell-free approach to arterial substitutes [154]. They investigated the grafting of porous tubular PGSeb scaffolds, coated with heparin and enclosed in a PCL sheath. Smooth muscle cells infiltrated the grafts modifying their mechanical properties: increased compliance and softness were observed and alterations in the stress-strain curves were noted. Laser Doppler ultrasound imaging of the grafts indicated synchronous pulsation with host aorta, which, according to the authors, is the first report of any vascular graft that pulses with host arteries. 

##### Bone and Cartilage Tissue Engineering

The elastic modulus of PGSeb scaffolds can match the equilibrium elastic properties of native cartilage and it was demonstrated that PGSeb scaffolds supported the formation of a cartilaginous matrix in vitro [103,137]. 

**Figure 9:**
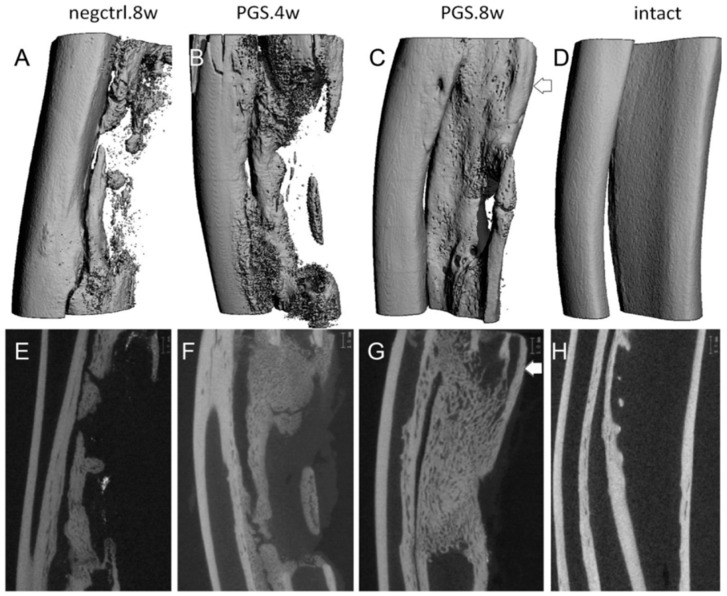
Micro computed tomography 3D rendering of four week (**B**) (*n* = 2) and eight week (**C**) (*n* = 5) retrieved ulna samples compared to an eight week empty negative controls (**A**) (*n* = 5) and an intact ulna (**D**). (**E**–**H**) The corresponding midsection X-ray for control, four week, eight week, and intact ulna. Reprinted from [155]. Copyright 2017, with permission from Elsevier.

PGSeb was also investigated for bone regeneration and the repair of critical-sized bone defects (defects that do not heal spontaneously during the lifetime of a subject). Although PGSeb is less stiff than native bone, its stiffness is representative of a developing skeletal tissue [155] and because of its elasticity it supplies a load-transducing environment, which is believed to be beneficial to cell differentiation and maturation. It was demonstrated that PGSeb without seeded cells [155] or seeded with osteoprogenitor cells [156] was able to facilitate the bridging of bone defects, as illustrated in Figure 9. 

**Figure 10:**
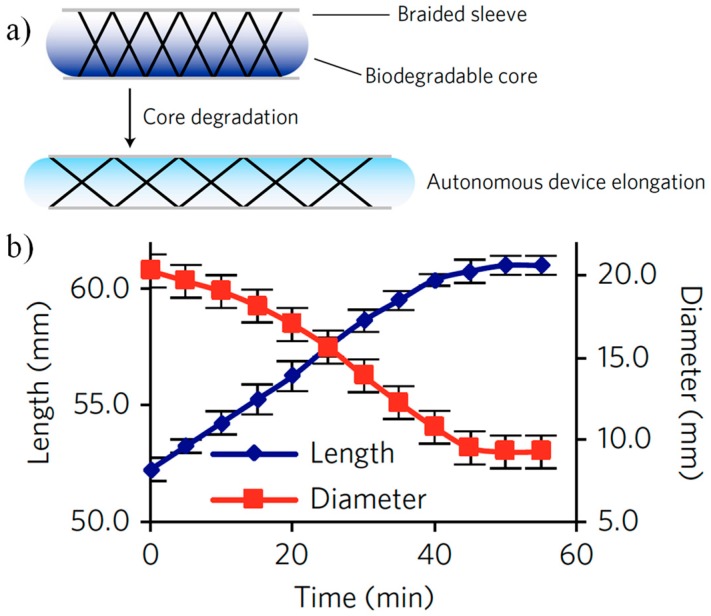
Growth-accommodating device concept. (**a**) Schematic of a degradable polymer core (dark blue) placed inside a braided sleeve to control sleeve diameter, coupling inner polymer degradation to braided sleeve elongation. (**b**) Braided sleeve behavior and core degradation [157]. Reprinted with permission from Springer Nature, copyright 2017.

Any implant for pediatric applications should be able to adapt and accommodate to the physiologic development of children. However, most implants have a fixed size, limiting their efficacy and often requiring multiple interventions. Feins et al. designed a device able to accommodate tissue growth, a critical characteristic for implants aimed at pediatric applications (Figure 10) [157]. The device was composed of a PGSeb biodegradable core and an outer braided sleeve and as the PGSeb degraded by surface erosion, the diameter of the inner core progressively decreased, resulting in an extension of the braided sleeve (Figure 10). A stiffer PGSeb, prepared by curing the prepolymer at 155 °C for 86 h, was necessary to efficiently restrict the initial elongation of the device. The device was successfully tested in vivo on a bone growth model, and a heart valve growth model, boding exciting developments in this field.

##### Other Tissues 

Neeley et al. reported the microfabrication by spin-coating of a porous PGSeb membrane for the delivery of retinal progenitor cells (RPCs) for retinal tissue engineering [123]. In vitro studies indicated that RPCs adhere to and proliferate on PGSeb. The elastomeric property of PGSeb allowed the polymer seeded with cells to be scrolled and transplanted via syringe injection, protecting the cells from shearing forces and reducing trauma to ocular tissue during transplantation [158]. Furthermore, Ghosh et al. showed that diseased photoreceptors in the retina can be selectively removed by retinal detachment induced by the implantation of the non-porous PGSeb membrane in the subretinal space [159]. 

PGSeb was found promising for the engineering of the temporomandibular joint disc [160] and the repair of chronic tympanic membrane (TM) perforations [105,161]. It was reported that 91% of TMs repaired with spool-shaped plugs of PGSeb had healed six weeks following implantation. 

Although the potential of PGSeb for neural reconstruction applications had been demonstrated early on [110], the construction of a pure PGSeb device for neural tissue engineering was reported much later [162]. The device combined three stimulating factors, a micro-patterned surface, a neurotrophic gradient membrane and seeding with Schwann cells, and it was demonstrated that neural stem cells could attach, proliferate, and differentiate successfully on this device. PGSeb was also evaluated for the repair of the transection of spinal cords [163]. 

#### 7.2.2. Drug Delivery

Sun et al. were the first to investigate the potential of PGSeb for controlled drug release [164]. PGSeb was doped with 5-fluorouracil (5-FU), one of the most commonly used anticancer agents, to fabricate 5-FU-PGSeb implants. 5-FU-PGSebs were prepared by adding 5-FU to the prepolymers, before the curing step. High concentrations of 5-FU tended to reduce the DP and increase the degradation rate of the implants. The in vitro release profiles of 5-FU-PGSeb samples exhibited a biphasic release with an initial burst on the first day and almost 100% cumulative release of 5-FU in 7 days. 5-FU release rate was shown to decrease with increasing curing temperature [165]. In order to delay the release of 5-FU for applications requiring a longer time frame, 5-FU was derivatized and chemically conjugated with the glycerol units of PGSeb through ester bonds and PGS-5-FU-CH_2_COOH, was prepared [166]. The drug release pattern of PGS-5-FU-CH_2_COOH polymers was significantly improved and the polymer demonstrated significant in vitro cytotoxicity against tumor cells. This approach was further applied to curcumin [167] and a mimetic of brain derived neurotrophic factor, which enhanced the calcium transients of cardiomyocytes cultured on the modified PGSeb [147]. 

Louage et al. reported the preparation of PGSeb nanoparticles for drug delivery applications [144]. Two hydrophobic drugs with anti-mitotic activity, paclitaxel (PTX) and flubendazole, were encapsulated in PGSeb nanoparticles and successfully released. The PTX/PGSeb nanoparticles showed comparable results to commercial PTX formulations. Yang et al. used swelling drug load to charge PGSeb with berberine and chlorhexidine: PGSeb was swelled in ethanol to allow a better diffusion of the drugs and increased permeability [168]. Drug loading had no influence on the mechanical properties of PGSeb (Young’s modulus and strain at break) but it did affect the surface wettability. The berberine-loaded PGSeb performed well in anti-microbial and anti-bacterial tests and PGSeb loaded with berberine and chlorhexidine was found to be a good candidate for the treatment of periodontal disease.

PGSeb elementary osmotic pumps for the delivery of ciprofloxacin-HCl (CIP) have also been fabricated and evaluated for the development of antibiotic releasing devices for the treatment of chronic prostatitis [169]. The rapid in vivo degradation of PGSeb is expected to limit the use of this device to short-term drug therapies. Jen et al. investigated the immobilization and release of lentiviruses on PGSeb in the context of substrate-mediated gene delivery [170]. Finally, Lee and his colleagues reported the use of a PGSeb scaffold for the delivery of stromal cell-derived factor-1α (SDF-1α) embedded in a heparin/poly(ethylene argininyl aspartate diglyceride) coacervate [171]. 

#### 7.2.3. Other Applications

Pryor et al. showed that PGSeb barrier films were efficacious in reducing visceroparietal peritoneal adhesions in the rat cecal abrasion and peritoneal wall defect model, envisioning that PGSeb films may offer adhesion prevention in open and laparoscopic abdominopelvic operations [172]. Bountry et al. used PGSeb as the elastic dielectric component in pressure sensor patches, which exhibited higher sensitivities compared to the ones previously described in the literature and an improved response time [173]. A more complex sensor combining PGSeb and poly(octamethylene maleate (anhydride) citrate) was further developed to monitor tendon healing and allow for a personalized treatment [174]. 

A low crosslink density PGSeb was successfully used to coat mesoporous bioactive glass scaffolds [175]. The coated scaffolds exhibited enhanced toughness, an easily adjustable, wide range of mechanical strengths and very promising results in view of applications in bone tissue engineering. Finally, PGSeb was successfully coated by electrospray on nitinol stents for the treatment of varicose veins disease [176]. 

## 8. Poly(Glycerol Lactide), PGL

### 8.1. Synthesis 

Poly(lactic acid) is a biodegradable and bioactive thermoplastic aliphatic polyester derived from renewable resources, such as corn starch, cassava roots, chips or starch, or sugarcane. It is one of the most important bio-based polyester and in 2010 it was the bioplastic with the second highest volume consumption in the world. High molecular weight PLA can be synthesized via traditional ring-opening polymerization of lactide (Lact). Recently, more attention has been drawn to the direct melt copolycondensation of lactide with other multifunctional monomers to produce star or hyperbranched polymers, in order to improve some of its properties such as the poor hydrophilicity, the low cell affinity, and the low hydrolysis rate. 

Arvanitoyannis et al., synthesized three-arm star shaped poly(L-lactide) (PLLA) via ROP using glycerol as the core, producing a polyester with good biodegradability, low crystallinity, low melt viscosity and high molecular weight [177]. As a result of these properties, PLLA with a Gly core and its derivatives were used as drug delivery carriers, tissue engineering scaffolds and medical device materials. Polymerization of L-lactide (L-lact) with various amounts of Gly (100:1 up to 3:1 molar ratios) was carried out in a glass ampoule in the presence of stannous octoate (Sn(Oct)_2_) or tetraphenyl tin (TPT) as catalysts at 130 °C for 4 days. From GPC it was found that the molecular weight in L-lact/Gly polyesters decreased by reducing the molar ratio from 100:1 to 3:1, Table 7. The polymers synthesized using Sn(Oct)_2_ as a catalyst gave monodisperse curves while those synthesized with TPT bimodal curves. Almost all polyesters were semicrystalline materials except those prepared with L-lact/Gly ratios of 10:1 and 3:1. T_g_ and T_m_ temperatures for semicrystalline polymers decreased as the monomer ratio was reduced from 100:1 to 20:1 (Table 8). Enzymatic hydrolysis studies at pH 7.0 using *Rhizopus arrhizus* lipase demonstrated that all polyesters were degradable and that those with high Gly content and low percentage of crystallinity had the highest degradation rate. Similar three arm polymers were prepared by Luo et al. using lactic acid (LaA) and glycerol with different molar feed ratios (LaA/Gly = 20:1, 40:1, 60:1, 80:1, 100:1, 120:1, 140:1) by melt polymerization at a certain temperature, (140–180 °C), a pressure of 70 Pa, for 4–12 h [178]. The influence of different catalysts (SnO, SnCl_2_, ZnCl_2_, p-toluenesulfonic acid (TSA) and ZnO) on the reaction was evaluated. TSA gave polyesters with the lowest intrinsic viscosity (IV). For the other catalysts it was found that the highest viscosity was obtained for catalyst concentrations till 0.5 wt %. Reaction temperature played also an important role and it was found that at 140–160 °C polyesters with white color and the highest IV were obtained while at 170–180 °C lower IV was recorded and a yellowish color was observed due to decomposition. At such low polycondensation temperatures, reaction times up to 8 h are ideal and LaA/Gly molar ratios ranging from 60:1 to 140:1. In these conditions, semicrystalline material with the highest molecular weight were produced. These polymers had T_g_ values ranging from 35 to 41 °C and melting points ranging from 100 to 134.1 °C (Table 8). 

**Scheme 7:**
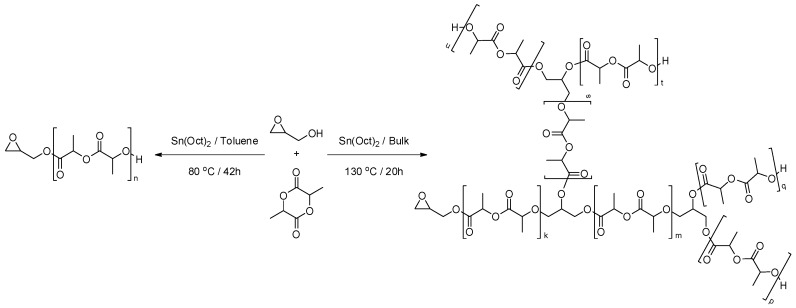
Synthesis of linear or branched glycerol-PLA polyester (PGL) from glycidol and lactide [180].

According to the above-described procedures, only three-arm polymers between glycerol and lactide could be prepared. In a different approach, hyperbranched glycerol-PLA polyesters (PGL) were synthesized starting from glycidol (Gld), a cyclic derivative of glycerol, by cationic ROP (Scheme 7). l-Lactide was used as a monomer and its polymerization with glycidol took place in bulk, at 100–110 °C, slightly above the melting point of l-lactide (95–96 °C), in the presence of boron trifluoride diethyl etherate (BF_3_·Et_2_O) or diphenyl phosphate as catalysts [179]. In this case the -OH group of glycidol initiates the reaction with lactide and polymerization continues with the subsequent opening of the oxirane ring of glycidol. A semicrystalline macromer with T_m_ 123–124 °C was produced, which led gradually to branched macromolecules with high molecular weight consisting of randomly dispersed PLLA and glycerol units. These polymers differed significantly in their form and properties compared to the star polymers that were prepared from glycerol and lactide. For their synthesis, several l-lact/Gld molar ratios were tested as well as different reaction conditions (times and temperatures). A general trend of increased molecular weight with increasing Lact/Gld molar ratio was reported by Pitet et al. using Sn(Oct)_2_ as catalyst in bulk polymerization at 130 °C [180]. Mixtures of branched structures were obtained with three-arm structures but also important amounts of polymers with higher DB. All branched copolymers were amorphous with T_g_ temperatures ranging between 47–54 °C, which were lower than the T_g_ values of linear PLA (about 60 °C). T_g_ temperatures increased when increasing the Lact/Gld ratio and thus the final molecular weight. 

**Figure 11:**
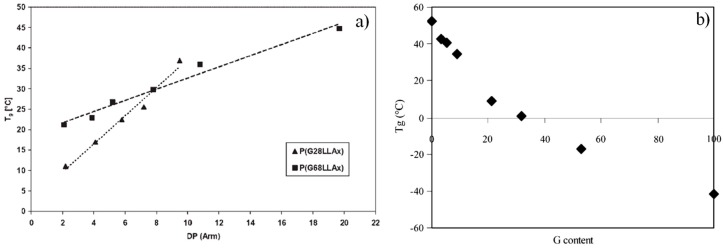
Correlation of the T_g_ of PGL block copolymers with: (**a**) the lactide content (expressed as the arm length). Reprinted with permission from [182]. Copyright 2007 John Wiley and Sons. (**b**) The glycerol content. Reprinted with permission from [181]. Copyright 2009 Elsevier Ltd.

In a similar work a series of hyperbranched lactide/glycidol copolymers of various compositions were synthesized (including 95:5, 90:10, 80:20, 70:30, and 50:50 Lact/Gld feed ratios), by ring-opening polymerization in bulk process at 130 °C (10–12 h reaction) using various catalysts such as tin octoate, magnesium diethoxide, and tetraphenyl tin [181]. Among them it was found that Sn(Oct)_2_ is the most effective in all studied compositions producing copolymers with the highest M_w_. It was once more confirmed that the molecular weight and T_g_ values increased by increasing the Lact/Gld ratio, (Figure 11b). The copolymers obtained from the highest Lact/Gld ratios (90:10 and 95:5) were semicrystalline materials (T_g_ values: 34.7 and 42.5 °C and T_m_ values: 91.9 and 127.6 °C, respectively). Enzymatic hydrolysis studies, using proteinase K at 37 °C for 24 h, demonstrated that copolymers with a low glycidol content (<5 wt %) had the highest hydrolyzability (up to 82%) while above 10 wt % glycidol the hydrolyzability remained stable (about 10%). Copolymers with glycidol content higher than 20% started to become water-soluble and those with a Lact/Gld 50:50 ratio were completely soluble. 

When glycidol and lactide are used as monomers, as discussed above, PGL copolymers consisted mainly of randomly dispersed Lact and Gly units. This is because both monomers react simultaneously during bulk polymerization. Multi-arm star block copolymers can be also prepared from a hyperbranched polyglycerol block and poly(l-lactide) arms. These copolymers have been prepared using HBPG oligomers as initiators instead of glycerol or glycidol. In such cases, HBPG core should be first prepared and, in a second stage, l-lactide is added in different ratios, in order to produce PLLA arms with different molecular weights [182,183]. Ring opening polymerization was carried out using Sn(Oct)_2_ as catalyst at 115 °C for 24 h. It was reported that the molecular weight increased by increasing the amount of lactide. Additionally, starting from higher M_w_ macroglycerol, seemed to produce multi-arm copolymers with higher M_w_. A similar copolymer was produced using the same monomers but different conditions (125 °C, 1–4 h [184,185] or 195 °C in the presence of Sn(Oct)_2_ [186]). Gottschalk et al. obtained amorphous materials with a single T_g_, which increased gradually by increasing the length of the PLLA arms and ranged between 11 to 45 °C (Figure 11a) [182]. Similar amorphous materials were prepared by Gardella et al., by the ROP of d-lactide (d-lact) with HBPG as a multifunctional macroinitiator and Sn(Oct)_2_ as a catalyst, in bulk at 120 °C [187]. In DSC, only the T_g_ of poly(d-lactide) (PDLA) was observed, at about 41 °C. These hyperbranched compounds were used to prepare blends with PLLA to prepare electrospun fibers with enhanced modulus. On the other hand, Ouchi et al. reported that semicrystalline copolymers could be prepared from l-lactide and polyglycidol (PG) (monomer/OH ratio: 10, 20, 30, and 50) with T_g_ (35.6–41.1 °C) and T_m_ (119.0–167.5 °C) values that both increased with increasing lactide content [183]. This was expected since PLLA would form longer arms. These results are in good agreement with the findings of Petchsuk et al. who observed that molecular weight increased by increasing the d-lact/PG ratio from 5:1 to 50:1 and reported that semicrystalline materials were prepared with T_g_ (36–46 °C) and T_m_ (90–140 °C) both increasing by increasing the d-lact/PG ratio [188]. 

Instead of ring opening polymerization, similar hyperbranched amphiphilic copolymers, consisting of PLA and HBPG blocks, were synthesized in solution, from the coupling of amino-HBPG (HBPG-NH_2_) with PLA, in the presence of 1-ethyl-(3,3-dimethylaminopropyl) carbodiimide hydrochloride (EDC·HCl) [189]. The advantage of this method was that the all PLA arms had the same molecular weight, which was chosen from the beginning of the reaction. Biocompatible copolymers with different M_n_ were prepared and it was observed that the release rate of quercetin, a poorly water-soluble drug, decreased by increasing the M_n_ of the copolymers. 

**Scheme 8:**
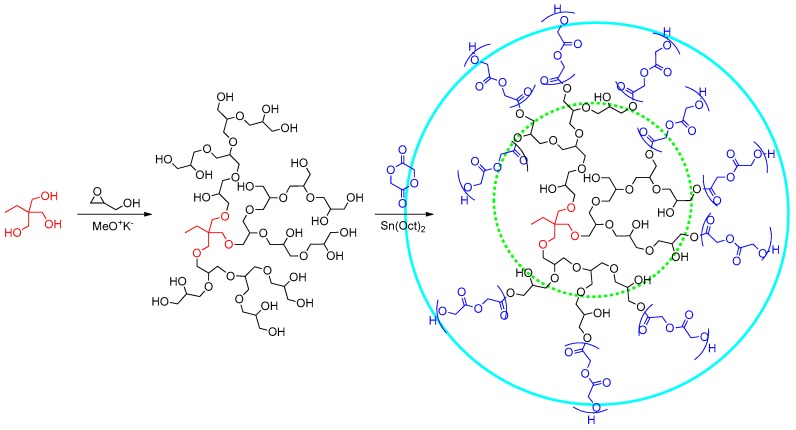
Synthesis of hyperbranched polyglycerol (HBPG)-PGA from glycidol and lactide [190].

Similarly to poly(glycerol lactide), polymers with polyglygolide were also synthesized. Polyglycolide based multi-arm structures using multifunctional HBPG as macroinitiator and the tin(II) 2-ethylhexanoate catalyzed ROP of glycolide in the melt were reported (Scheme 8) [190]. It was found that by increasing the glycolide content, copolymers with higher molecular weights as well as with increased length of PGA arms, were obtained. These copolymers were semicrystalline with T_g_ values of 10–18 °C and T_m_ ranging between 170 and 190 °C.

HBPG–PLGA copolymers were also be synthesized by esterification instead of ROP. Accordingly, HBPG was dissolved in anhydrous DMF and a suitable amount of PLGA was added, EDC and N-hydroxysuccinimide acted as esterification catalysts [191]. The reaction was completed in 48 h at room temperature. These copolymers could form nanoparticles, which were functionalized with transferrin antibody (OX26) and were used for the brain delivery of endomorphins. Cellular uptake study showed that the uptake of nanoparticles by the brain microvascular endothelial cells was both time- and concentration-dependent while OX26-modified nanoparticles achieved better analgesic effects than free endomorphins.

### 8.2. Applications 

Almost all applications of hyperbranched polyglycerol-poly(lactic acid) copolymers concern matrices for controlled drug release. Amphiphilic HBPG-PLA conjugates were prepared using HBPG-NH_2_, which was coupled to PLA in the presence of EDC·HCl and hydroxybenzotriazole as coupling agents, and were used for BSA protein delivery [192]. In aqueous solution the copolymer self-assembled into nanoparticles. It was non-toxic, biocompatible and biodegradable and exhibited a high BSA encapsulation capacity. It was found that the protein release rate could be adjusted from the PLA molecular weight in the hyperbranched copolymers. Similar hyperbranched amphiphilic copolymers (HBPG–PLA–PEG–FA), containing PEG and folic acid (FA) have been used to achieve cell targeting properties [193]. Paclitaxel was nanoencapsulated in these copolymers and it was found that the PTX loaded nanoparticles exhibited enhanced inhibition on folate receptor positive tumor cells due to the folate-mediated targeting. 

**Figure 12:**
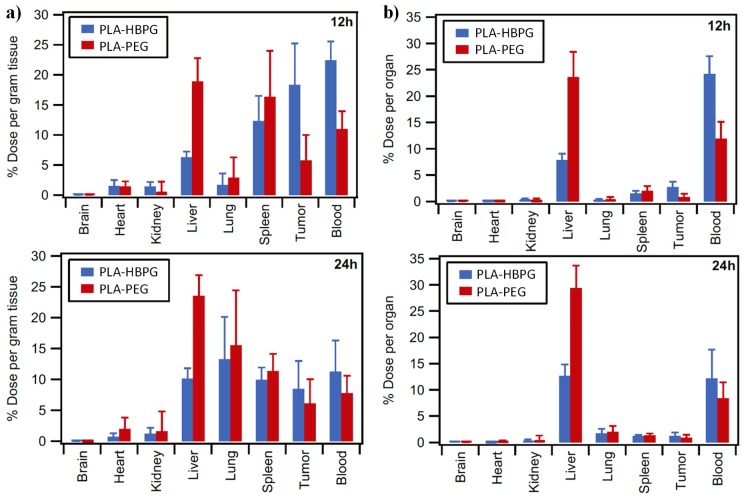
Biodistribution of 1,10-dioctadecyl-3,3,30,30-tetramethylindodicarbocyanine, 4-chlorobenzenesulfonate salt loaded PLA-HBPG and PLA-polyethylene glycol (PEG) NPs at 12 h and 24 h after intravenous administration, recorded as (**a**) percent dose per gram tissue, and (**b**) percent dose per organ. Data are shown as mean ±SD (*n* = 6). Reprinted from [194]. Copyright 2014 Elsevier Ltd. All rights reserved.

**Figure 13:**
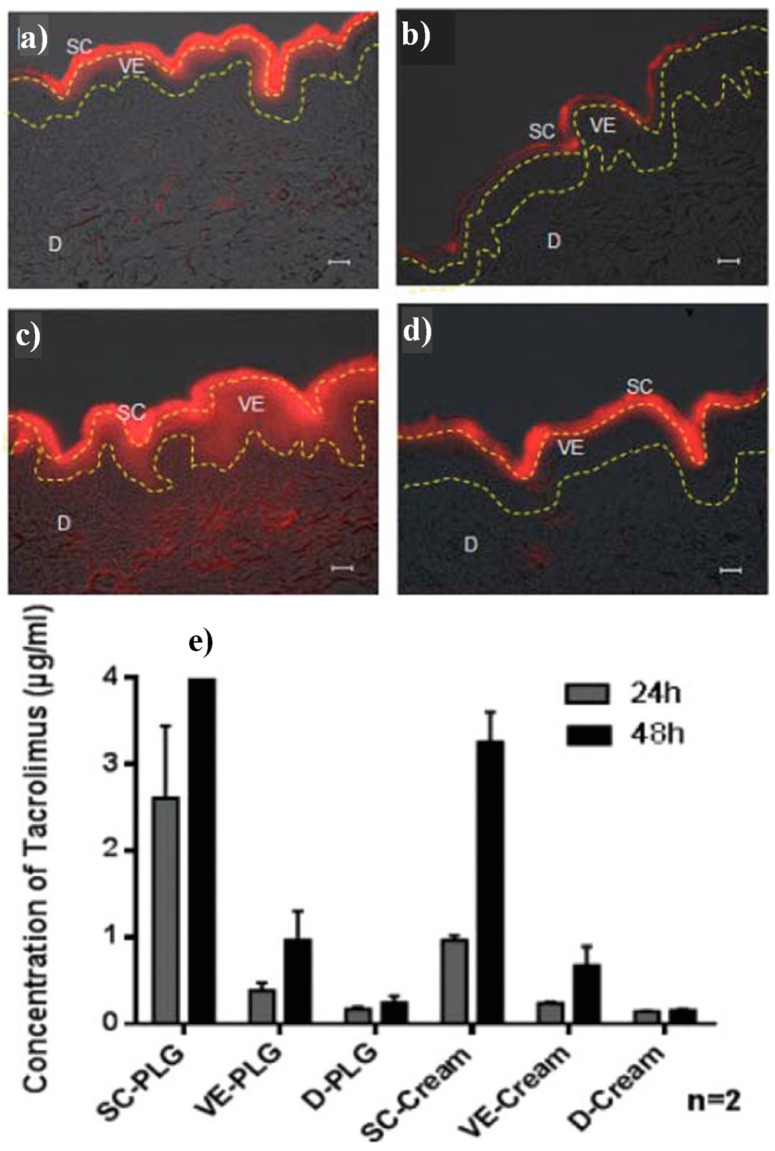
Representative overlay microscopy images of bright field and fluorescence human skin. (**a**,**c**) Nile red-loaded PLG (0.001% *w*/*w*). (**b**,**d**) Nile red incorporated into base cream (0.001% *w*/*w*). (**e**) Dermal distribution of tacrolimus (TAC) delivered by PLG and cream (TAC, 0.03% *w*/*w*) in different layers of human skin after 24 h and 48 h incubation times. Layers of skin: SC: stratum corneum, VE: viable epidermis, D: dermis. Reproduced from reference [196] with permission from The Royal Society of Chemistry.

Nanoencapsulation of camptothecin, another anti-tumor drug, was done in hyperbranched PLA-HBPG copolymers and was compared to PLA–PEG copolymers [194]. It was found that despite being similar in size, drug release profile and in vitro cytotoxicity, the PLA-HBPG NPs showed significantly longer blood circulation and significantly less liver accumulation than PLA–PEG copolymers (Figure 12). Similarly, copolymers consisting of a PLA core and a HBPG shell modified with adenosine, were used to form nanoparticles that could cross the blood–brain barrier [195]. Due to their peculiar solubility, Zabihi et al. used hyperbranched PGL to prepare nanoparticles in aqueous solutions for the delivery of tacrolimus (TAC), a hydrophobic drug [196]. Nanoparticles of 35 nm (average size) were prepared and high TAC loadings (14.5% *w*/*w*) were obtained. PGL nanoparticles significantly increased the penetration of Nile red into the viable epidermis and dermis layers of skin (Figure 13). TAC diffusion into different layers of skin was time-dependent and the PGL delivery system resulted in 1.74 × higher stratum corneum, 1.44 × viable epidermis, and two-times increased TAC levels in the dermis, respectively, compared to the cream reference. An additional advantage of these copolymers was that they did not have significant cytotoxicity against primary human fibroblasts and keratinocytes. 

**Scheme 9:**
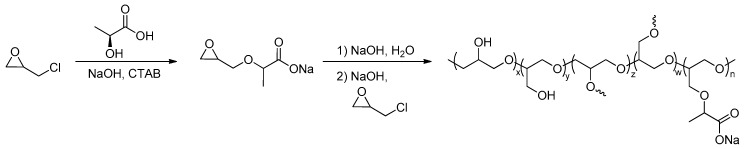
Synthesis of PGly-PLLA [199].

Drug release is affected by many characteristics of PGL copolymers, but mainly by their molecular weight. In a recent study, HBPG-PLLA hyperbranched copolymers were synthesized at 155 °C for 18 h using HBPG with M_n_ 500 and 2500 g/mol, L-lactide and Sn(Oct)_2_ [197]. The molecular weight of the prepared copolymers depended on the M_n_ of HBPG and the amount of lactide and increased when both of them increased. These copolymers formed self-assembled core-shell micelles in water or organic solvents and acetylsalicylic acid was encapsulated in them. Its release rate was controlled by the M_n_ of the copolymers, and decreased by increasing it. Similar results were also reported by Mohammadifar et al., when Rose Bengal, used as a model dye, was encapsulated in HBPG–PLA–PCL polymers comprised of a HBPG core and PLA-PCL shells [198]. The amount of encapsulated dye and its release depended on the molecular weight of the HBPG core as well as the thickness and type of shell. Controlled release of the dye was achieved. Novel pH-responsive polyglycerol (PGly)-based hydrogels were successfully synthesized through the reaction of epichlorohydrin with l-lactic acid (LLA) in the presence of sodium hydroxide (NaOH), and cetyltrimethylammonium bromide as a phase transfer catalyst, followed by hydrolysis, and finally polymerization and crosslinking (Scheme 9) [199]. These copolymers were used for drug release at different pH. Finally, HBPG–PLA with a polyglycerol core and PLA side chains was used to physically modify polyethylenimine for several applications [200]. 

## 9. Poly(Glycerol ε-Caprolactone), PGCL

Poly(ε-caprolactone) (PCL) is a biocompatible, semicrystalline, water-insoluble polymer which has gained much attention owing to its amicable nature and tailorable properties. It has been extensively investigated for various applications which include drug delivery, scaffolding and tissue engineering. However, PCL has high degree of crystallinity (50–60%) and a slow in vivo hydrolysis. Star and hyperbranched copolymers have been synthesized using multifunctional co-monomers, like glycerol, for medical and biological applications [201,202]. As illustrated earlier with poly(glycerol lactate), star-shaped polymers have specific properties related to their structure that differentiate them from their linear counterparts and thus star-shaped poly(ε-caprolactone)s have garnered significant attention. 

### 9.1. Synthesis and Properties

#### 9.1.1. Three-Arm Poly(Glycerol ε-Caprolactone)

**Scheme 10:**
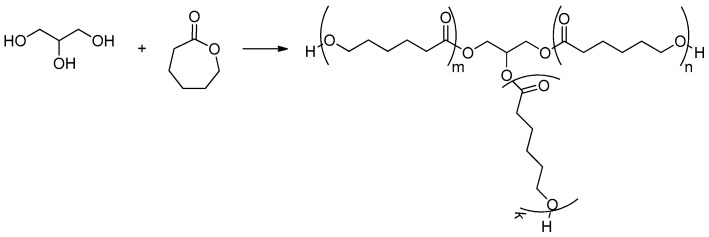
General synthesis of three-arm poly(glycerol ε-caprolactone).

A typical procedure to obtain star-shaped PCLs is the catalyzed ring-opening polymerization of caprolactone, in the presence of a multi-functional alcohol (glycerol, trimethylolpropane, pentaerythritol, etc.). When glycerol is used to initiate the polymerization, a three-arm poly(glycerol ε-caprolactone) (PGCL) polyester is produced (Scheme 10).

For PGCL synthesis the most often used catalyst has been tin(II) 2-ethylhexanoate or stannous octoate, Sn(Oct)_2_ [203,204,205,206,207,208,209,210,211], however, acidic (BF_3_·O(CH_3_)_2_) [212,213], and enzymatic (Novozym 435) [214,215] catalysis have been explored as well. The polymerization has been carried out between 110 °C and 130 °C when Sn(Oct)_2_ was used (about 48 h polymerization) or at lower temperatures when Novozym 435 (70 °C) or BF_3_O·(CH_3_)_2_, (80 °C) were used. The PGCL polymers were characterized by GPC [203,206,207,208,209,210,212,213,214,215], FTIR [203,207,209,210,215], NMR [203,206,207,208,209,210,212,213,214,215], DSC [203,206,212,214,215], and X-ray diffraction [206,215]. 

PCL-glycerol polyesters were synthesized in one step in the presence of Novozym 435 under mild reaction conditions (70 °C) and the effect of several parameters such as reaction time, glycerol to ε-caprolactone (CL) ratio and CL to toluene ratio were studied [215]. Semicrystalline polymers exhibiting an enhanced hydrophilicity compared to neat PCL were obtained. The melting temperature, T_m_, of PCL was reduced from 54 to 48 °C with the incorporation of glycerol. Increasing the Gly content resulted in a M_n_ decrease (ranging from 1500 to 3100); M_n_ increased for reaction times up to 6 h and a reduction was observed at 8 h (Table 8). These results agree with the findings of Xue et al., who used a similar enzymatic procedure (dioxane, Novozym 435 at 70 °C) [214]. PGCL with T_m_ of 45–47 °C were obtained by controlling the M_n_ between 2720 and 4200 g/mol. 

Star shaped PGCL polymers were also prepared using stannous octoate as a catalyst, via a melt procedure. A linear dependence of M_n_ on the CL/Gly molar ratio was observed and therefore the molecular weight of PGCL could be controlled by adjusting the molar ratio of the monomers [203,206]. In the study of Lang et al., 5:1, 10:1, 20:1, and 40:1 feed molar ratios of CL to the hydroxyl groups of glycerol were used and polymerization was carried out at 130 °C for 48 h in the presence of stannous octoate as catalyst [203]. The synthesized polymers were semicrystalline with melting points ranging between 40 to 50 °C. T_m_ as well as the degree of crystallinity both increased with increasing molecular weight. Wide-angle X-ray diffraction curves showed similarities in the crystal structures of the star-shaped PGCL and linear PCL [206]. However, the degree of crystallinity decreased when increasing the number of arms.

BF_3_, which is a strong Lewis acid, can also initiate the ring opening polymerization of ε-caprolactone via a cationic mechanism [213]. Gly (0.018–0.073 mol/dm^3^) and CL were polymerized at 80 °C for 72 h using BF_3_ (0.0095 mol/dm^3^) as catalyst. It was found that M_n_ decreased by increasing the Gly concentration, even though monomer conversion had the opposite trend. Very broad molecular weight distributions (MWD) were obtained, due to the catalyst. In contrast, when Sn(Oct)_2_ was used as catalyst a narrow MWD was obtained and M_n_ increased by reducing the Gly content (vide supra) [206]. 

**Scheme 11:**
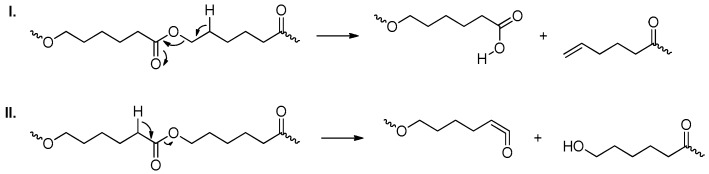
Mechanisms I and II for the thermal degradation of PGCL.

PGCL exhibited a high thermal stability, which could be improved not only by increasing the molecular weight but also by increasing the number of arms [208]. The thermal degradation of PGCL occurs through two different mechanisms (Scheme 11). Initially, the ester bonds of the PCL chains are pyrolyzed into alkene and carboxyl functional groups (mechanism I). In the second stage, mechanism I takes place concomitantly with the cleavage to ketene and hydroxyl groups (mechanism II). Enzymatic degradation of star-shaped PCLs that had the same molecular weights (M_n_ = 10,300) but different arm numbers showed that the biodegradation rate of star-shaped PCL with one to three arms increased with increasing arms number [206]. However, a further increase of the arms number from three to five resulted in a decreased biodegradation rate.

#### 9.1.2. Synthesis of Block Polyglycerol/Poly(ε-Caprolactone) Copolymers

**Scheme 12:**
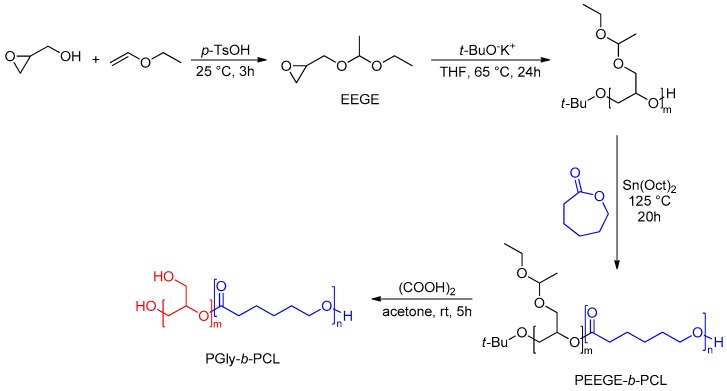
Synthesis of linear poly(glycerol)-poly(caprolactone) block copolymers [216].

In the above-mentioned works, we discussed three-arm copolymers, however other architectures such as linear or multi-arm/hyperbranched copolymers, have also been synthesized. Jun et al. synthesized linear PGCL using glycidol, instead of glycerol, which was reacted with ε-caprolactone. In this case, in order to avoid the formation of multi-arm polymers, the hydroxyl group had to be initially protected, and the copolymers were prepared in a three-stage procedure (Scheme 12) [216,217,218]. Ethoxyethyl glycerol ether (EEGE) was synthesized initially from glycidol and ethyl vinyl ether using p-toluenesulfonic acid as a catalyst. In the next step, EEGE was polymerized at 65 °C in THF for 24 h using potassium tert-butoxide as an initiator. Poly(ethoxyethyl glycerol ether) (PEEGE) was further copolymerized with ε-caprolactone at 125 °C for 20 h using stannous octoate. Finally, deprotection of the -OH group with oxalic acid afforded PG-*b*-PCL. This copolymer was semicrystalline with T_m_ 55 °C and was used as an emulsifier to stabilize lipiodol nanoemulsions (vide infra). 

As discussed with poly(glycerol lactide), core multi-shell polymers based on hyperbranched polyglycerol (HBPG) have recently been synthesized for potential biomedical applications. Self-assembly and solubility behavior as well as loading capacity of core-shell nanocarriers can be controlled by manipulation of molecular weight and type of core and shells. Multi-arms copolymers were synthesized by the ROP of CL in the presence of polyglycerol (PGly) initiator and Sn(Oct)_2_ catalyst, yielding oligomers with hydroxyl end-groups [219]. 

Diblock hyperbranched amphiphiles were prepared by the reaction of HBPG, obtained by the adenine-initiated anionic ROP of glycidol, and CL [220]. The HBPG-PCL copolymers were further reacted with citric acid to obtain adenine–polyglycerol–poly(ε-caprolactone)–polycitric acid triblock hyperbranched-linear-hyperbranched copolymers. This system self-assembled in aqueous solutions, producing nearly monodispersed nanoparticles with an average size of 120 nm. The encapsulation and release of curcumin was studied to demonstrate the potential of these copolymers for drug delivery applications. Triblock copolymers consisting of a HBPG core, a PCL intermediate shell and a poly(ethylene glycol) (mPEG) outer shell, have also been synthesized [221]. According to the reported procedure, linear monohydroxy-terminated diblock copolymers were synthesized by the ROP of CL using mPEG as a macroinitiator and Sn(Oct)_2_ as a catalyst. The resulting -OH end groups were further reacted with succinic anhydride to be modified to -COOH groups. In the last step, the -COOH groups of the diblock copolymer were coupled to HBPG–NH_2_ to yield the final material. The triblock copolymer formed core multi-shell nanoparticles and the anti-inflammatory drug dexamethasone was loaded into these. Skin penetration studies demonstrated that these nanocarriers were safe as skin penetration enhancers.

**Scheme 13:**
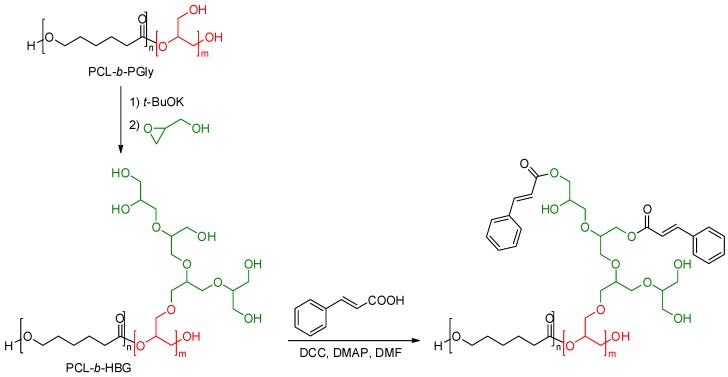
Synthesis of PCL-HBPG block copolymers [224].

**Figure 14:**
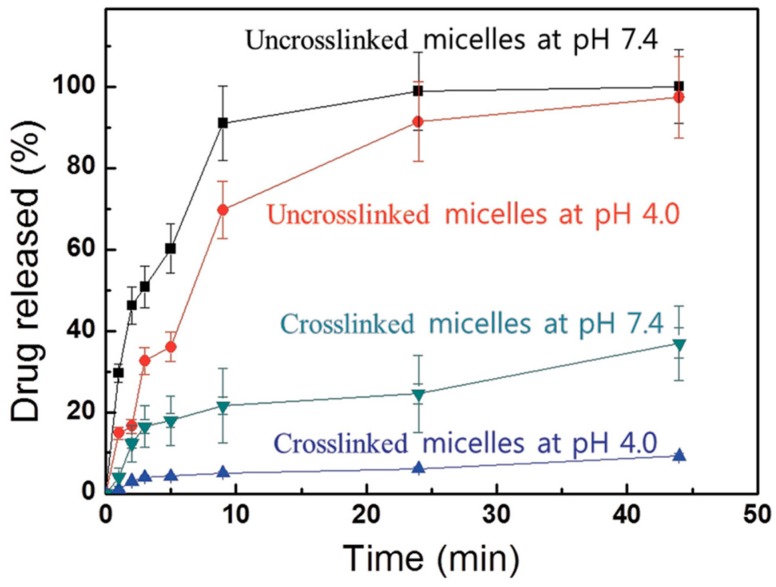
Release profiles of ibuprofen from the PCL-*b*-hbPG-CA micelles at 37 °C; at pH = 7.4 without UV crosslinking (■), at pH = 4.0 without UV crosslinking (●), at pH = 7.4 after UV crosslinking (▼), and pH = 4.0 after UV crosslinking (▲), respectively. Reprinted with permission from reference [224]. Copyright 2015, John Wiley and Sons.

**Scheme 14:**
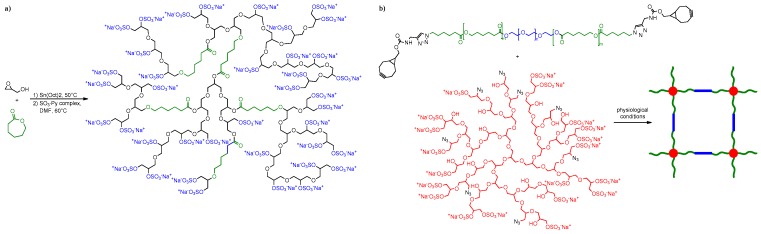
(**a**) Synthesis sulfated HBPG-PCL random copolymer [226]. (**b**) Hydrogel consisting of sulfated dendritic polyglycerol units linked with PCL–PEG–PCL arms via a strain promoted azide–alkyne cycloaddition reaction [228].

The “reverse” copolymers consisting from a PCL core and an HBPG shell have been also mentioned in literature. Four-arm PCL was initially synthesized and was further reacted with glycidol in basic conditions (potassium methylate solution) [222]. The resulting amphiphilic PCL hyperbranched polyglycerol derivative was studied as a matrix for drug and gene delivery applications. Similar linear poly(ε-caprolactone)-click-hyperbranched polyglycerol (PCL-click-HBPG) amphiphilic copolymers were synthesized by the “click” reaction of linear PCL-N_3_ with hyperbranched HC≡C-HBPG-Br [223]. These polymers were used to fabricate membranes by phase inversion and it was found that the HBPG content in the PCL-click-HBPG copolymers could be used to control the pore size and porosity of the membranes. A similar block copolymer, consisting of a hydrophobic PCL block and a hydrophilic hyperbranched polyglycerol block, was synthesized starting from linear block PGly-PCL copolymers [224]. Block PGly-PCL was synthesized according to Scheme 13 [218] and, PGly hydroxyl groups were further reacted with glycidol to produce the hyperbranched polyglycerol structures (Scheme 14). 4-hydroxycinnamic acid was covalently added and the copolymer was finally UV crosslinked. These block copolymers formed spherical nanoscale micelles, in which ibuprofen was encapsulated. Release studies demonstrated that drug release is slowed down by crosslinking (both at pH 4 and pH 7), allowing for a better control over the release rate (Figure 14). Finally, surface functionalized silica nanoparticles with PCL-HBPG block copolymers were prepared by successive ring-opening polymerizations (CL initially and then glycidol) [225]. It was found that these functional nanomaterials had a higher loading capability for small molecules than those with linear PEG. 

#### 9.1.3. Functionalized Polyglycerol/Poly(ε-Caprolactone) Copolymers

All the aforementioned copolymers with hyperbranched polyglycerol blocks bear surface hydroxyl groups, which increase the hydrophilicity of the final copolymer. However, these hydroxyl groups might not always be pertinent for specific applications and have been modified. 

Hyperbranched polyglycerols, containing caprolactone segments and bearing a sulfate outer shell were synthesized and evaluated for anti-inflammatory applications [226]. Glycidol and ε-caprolactone were initially copolymerized (50 °C, 4:1 Gld:CL molar ratio, Sn(Oct)_2_), and sulfation of the copolymers was performed in the presence of sulfur trioxide pyridine complex (1.5 eq/OH groups), in dry DMF, at 60 °C (Scheme 14a). The molecular weight of the copolymers could be controlled by tuning the polymerization conditions, the monomer to catalyst ratio, as well as the stirring and the time of the polymerization [227]. Compared to dendritic polyglycerol sulfate (dPGS), these sulfated copolymers had hydrolysable ester bonds and were biocompatible, while they exhibited a significant lower influence on blood coagulation and a higher inhibition of complement activity than heparin.

**Figure 15:**
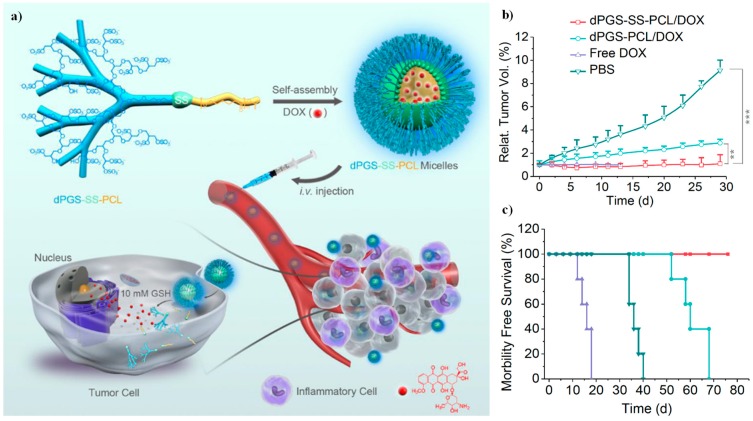
(**a**) Illustration of dendritic polyglycerol dPGS-SS-PCL micelles for active targeting to inflammation-related tumor tissues and triggered cytoplasmic drug release within tumor cells. (**b**) Tumor growth curves of MCF-7 human mammary carcinoma xenografted mice after treatment with dPGS-SS-PCL/DOX micelles, dPGS-PCL/DOX micelles, free DOX, or PBS. The drugs were administrated on day 1, 4, 7, 10, 13, and 16 (7.5 mg DOX equiv/kg) (*n* = 6). ** *p* < 0.01, and *** *p* < 0.001 (Student’s t test). **c**) Survival data for mice in different groups within a period of 76 d. Reprinted with permission from [229]. Copyright 2016 American Chemical Society.

A similar sulfonated injectable hydrogel, consisting of sulfated dendritic polyglycerol units connected with PCL–PEG–PCL arms has been recently reported (Scheme 14b) [228]. This is a biocompatible and highly degradable hydrogel that could be used for biomedical applications. The same group further reported the synthesis of a dPGS-SS-PCL amphiphilic block copolymer, with an M_n_ of 4.8−3.7 kg/mol and a negatively charged sulfonate surface, capable of self-assembling into small sized micelles (Figure 15) [229]. dPGS-SS-PCL block polymer was obtained via a copper-free strain-promoted azide−alkyne cycloaddition between linear PCL-SS-cyclooctyne (M_n_ = 3.7 kg mol^−1^) and dendritic azide-dPGS (M_n_ = 4.8 kg/mol, sulfation 88%). The synthesized copolymers had a low toxicity and organized into biodegradable micelles, that could shed their dPGS shells. The micelles were loaded with doxorubicin (DOX) and the loaded micelles exhibited a high tolerable dosage (more than 40 mg/kg) and a rather long plasma half-life (ca. 2.8 h), fluorescence imaging revealed an extraordinary tumor accumulation, and in vivo complete inhibition of tumor growth was achieved.

**Figure 16:**
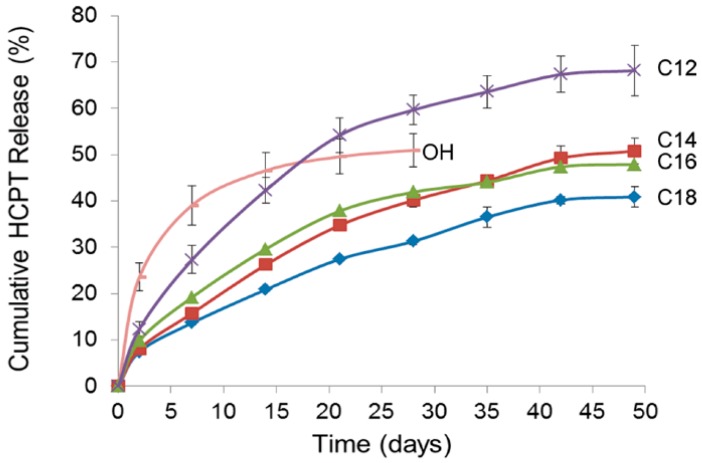
Cumulative release of 10-hydroxycamptothecin from drug loaded films in PBS at 37 °C (*n* = 3). Reprinted with permission from [230]. Copyright 2012 American Chemical Society.

As mentioned earlier for other aliphatic polyglycerol polyester copolymers, the secondary side hydroxyl groups can be esterified with acyl groups producing more hydrophobic materials with completely different properties. PGly-co-PCL random copolymers with side alkyl groups (C_12_, C_14_, C_16_, C_18_) were prepared via a three-step synthesis [230]. In the first step CL was copolymerized with 5-benzyloxy-1,3-dioxan-2-one in the presence of Sn(Oct)_2_. The secondary hydroxyl group of glycerol was then deprotected (H_2_/Pd removal of the benzyl group) [231] and, finally, the hydroxyl groups were esterified by the corresponding acids, in the presence of dicyclocarbodiimide. All copolymers were semicrystalline. Neat PGly-co-PCL had the lowest T_m_ (23 °C), the polymers with C_12_-C_16_ alkyl groups had T_m_ values 40–41 °C while the one with the C_18_ chain had a lower T_m_ (about 31 °C). These values are close to those mentioned for three-arm or block PGly-PCL copolymers [203,214,215,218]. 10-Hydroxycamptothecin drug was added in cast films and, it was observed that copolymers with increasing alkyl chain length had a progressively decreasing release rate (Figure 16). This was attributed to the increased hydrophobicity of these materials.

#### 9.1.4. Functionalized Polyglycerol/Poly(ε-Caprolactone) Copolymers with Additional Co-Monomers

More complicated structures of PGly-PCL copolymers have also been prepared using additional comonomers. Poly(glycerol-co-sebacate-co-ε-caprolactone) (PGSCL) elastomers were synthesized using glycerol, sebacic acid and ε-caprolactone (Gly/SeA/CL mole ratio 1:1:1), at a temperature of 120 °C, and in the presence of stannous octoate [232]. A wax-like prepolymer was initially synthesized and then further polymerized at 150 °C under vacuum (5 Torr) for 24 h. The T_g_ of the prepared elastomer was −36.96 °C, Young’s modulus was 46.08 MPa and the compression strength 3.19 MPa. Cell culture studies revealed that the prepared materials were biocompatible and thus could be used in tissue-engineering and drug-delivery systems.

Double-bond-functionalized three-arm poly(ε-caprolactone) was prepared in order to form hybrid amphiphilic networks [204]. PGCL was typically synthesized via the ROP of CL in the presence of glycerol, and the PCL chains were further functionalized with a double bond after reacting with maleic anhydride. This hydrophobic polymer was UV-crosslinked in the presence of a photo-initiator, with poly(ethylene glycol) diacrylate (PEGDA), a hydrophilic macromer, to form hybrid network hydrogels. The ratio of the two polymers was varied, affecting the hydrophilic/hydrophobic character of the network. Semicrystalline hydrogels were obtained, with a biphasic swelling behavior, consisting of an initial burst swelling and a plateau which was reached within 2–5 days. The burst swelling ratios (at 24 h) increased with an increase in PEGDA content. The in vitro release of bovine serum albumin from these hydrogels was evaluated and it was found that the hydrogels with the highest 3D porous network and hence the higher swelling ratio, gave the fastest and the more important BSA release. 

A novel smart drug delivery system for prolonged and sustained controlled drug release was developed by physically incorporating glycerol poly(ε-caprolactone)-based microspheres into a temperature sensitive poly(N-isopropylacrylamide) (PNIPAAm) hydrogel [205]. A biphasic system was thus prepared, composed of crystalline PGCL microspheres with T_m_ about 54–56 °C and amorphous PNIPAAm with a T_g_ about 145–152 °C. When ovalbumin was directly loaded into the PNIPAAm hydrogel it was released quickly. However, when it was loaded in the microspheres, the release rate dropped dramatically, and the release time was prolonged. Furthermore, owing to the surrounding PNIPAAm hydrogel a temperature-dependent (and controlled) drug release was observed.

Glycerol-caprolactone oligomers containing 1 or 2 caprolactone units were prepared by the ROP of ε-caprolactone with glycerol, at 130 °C, in the presence of stannous octoate and were further functionalized with acryloyl chloride (N(CH_3_)_3_, THF, N_2_ atmosphere, dark) [233]. The obtained polymers were low-viscosity liquids that were photopolymerized in the presence of a photo-initiator (camphorquinone) and a co-initiator (dimethylaminoethyl methacrylate) by exposure to 470 nm blue LED light. High degrees of conversion were achieved for the photopolymerization (>80% after 1 min curing). The crosslinked materials had a high compressive modulus (65.7 ± 12.7 – 80.9 ± 6.1 MPa) and they were destined to soft tissue engineering and medical 3D printing.

### 9.2. Applications

Likewise PGLA, the majority of applications of PGCL polyesters regard delivery systems, some were briefly mentioned above and some will be detailed in this part. Star-shaped PGCL oligomers were conjugated with norfloxacin [207], ciprofloxacin [209], and ofloxacin [210] to synthesize novel macromolecular prodrugs. Preliminary results showed that the release rate from depended on the kind of glycol unit in the polymer chain; the order of hydrolysis was as follows: PEG > Gly > pentaerythritol > dipentaerythritol [209,210]. Besides the kind of ester or glycol segment in the polyester, the release rate was also observed to be highly dependent on the pH of the medium. Three-arm PGCL polymers were used to prepare poly(ester-urethane)s which exhibited a shape-memory effect around body temperature and were appropriate for biomedical applications [214]. PGCL was also used for the nanoencapsulation of ropafenone, an anti-arrhythmic drug [234]. Increasing the Gly content, increased the hydrophilic character of the polymers and a higher drug release could be achieved. 

**Figure 17:**
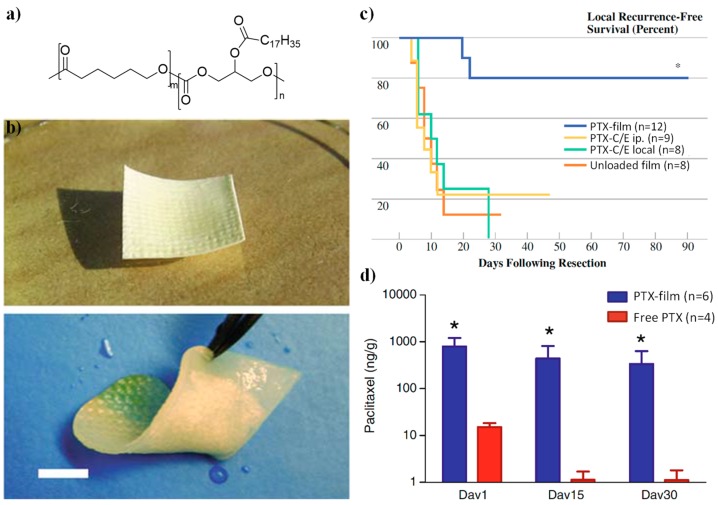
(**a**) Structure of poly(glycerol monostearate-co-ε-caprolactone) (PGCL-C_18_). (**b**) PGCL-C_18_ film on surgical collagen scaffold. The upper panel shows a dry film; the lower panel shows flexibility of a wet film. Scale bar, 5 mm. (**c**) Kaplan–Meier curve of freedom from local recurrence after tumor resection. After complete resection of the primary tumors, mice were randomly assigned to either local implantation of paclitaxel (PTX)-loaded film (300 μg paclitaxel per film), unloaded film, local subcutaneous injection of paclitaxel 300 μg in Cremophor/ethanol formulation (PTX-C/E local), or intraperitoneal injection of paclitaxel in Cremophor/ethanol formulation (PTX-C/E ip.). * Freedom from local recurrence in mice following implantation of PTX-loaded films was significantly greater than mice with unloaded films (*p* = 0.0001), PTX-C/E ip. (*p* = 0.0011), and PTX-C/E local (*p* = 0.0003). Reprinted from [238] with permission from Springer Nature, copyright 2009. (**d**) PTX-films result in significantly higher PTX concentration in local tissue than an intravenous injection of PTX in Cremophor/ethanol formulation, for at least 30 days. * *p* < 0.01 versus free PTX. Reprinted from [237] with permission from Springer Nature, copyright 2011.

PGly-*b*-PCL linear block copolymers exhibited an amphiphilic character and were used as emulsifiers to stabilize lipiodol nanoemulsions, in which paclitaxel anticancer drug was dissolved. Lipiodol, has a high iodine content and has been used as a clinically approved contrast agent for computer tomography (CT). The PGly surface of these nanoemulsions (100–200 nm) was further functionalized by a large number of folic acid for efficient tumor targeting and the nanoemulsions were loaded with 3% paclitaxel. In vivo studies demonstrated that all nanoemulsions showed excellent anticancer activity (LD_50_ < 3 ng/mL) [218]. The secondary hydroxyl groups of linear PGly-*b*-PCL were modified with stearic acid (C_18_ chain) and the resulting polymer was used to prepare electrospun drug-loaded hydrophobic meshes as implantable drug depots to prevent and treat locoregional recurrence in colorectal cancer patients [235]. These meshes were mechanically robust and flexible and thus were appropriate to be incorporated into surgical procedures/devices during colorectal surgery. It was found that the drug (CPT-11 or SN-38) was released over a period of time >90 days and significant in vitro tumor cytotoxicity against a human colorectal cell line (HT-29) was observed. It was also shown, with SN-38, that the copolymer-doped electrospun meshes exhibited significantly slower drug release relative to their melt controls [236]. Similar poly(glycerol monostearate-*co*-ε-caprolactone) copolymers have been prepared and loaded with paclitaxel drug, aiming at the preparation of carriers appropriate for local release to suppress tumor growth (Figure 17) [237,238]. It was found that implanted PTX-containing films prevented local tumor recurrence in vivo in 83.3% of animals, compared with unloaded films (12.5%). Although minimal paclitaxel remained in either plasma or tissue 10 days after a systemic injection, when paclitaxel was locally delivered via films (*p* = 0.024), the local paclitaxel concentration, at the site of surgical resection, was significantly greater (3000-fold) at 10 days.

## 10. Conclusions

Lately, a rising interest has been taken in star-shaped, multi-arm, and hyperbranched polymers. Compared to their linear counterparts, hyperbranched polymers exhibit a large number of terminal groups, higher solubilities, and lower viscosities (for comparable molecular weights), while they are altogether easily accessible substitutes for dendrimers and polyethers. In that context, aliphatic hyperbranched polyesters of glycerol which are a novel and versatile class of biodegradable and biocompatible polymers are emerging along traditional polyesters, such as PLA, PLGA, and PCL. 

Poly(glycerol sebacate) is by far the most studied polyester of this class, but poly(glycerol adipate) and poly(glycerol succinate) have been systematically studied as well. In stark contrast, poly(glycerol glutarate), poly(glycerol suberate), and poly(glycerol azelate) have been rather neglected, while poly(glycerol malonate) and poly(glycerol pimelate) have not yet been prepared. 

Varied synthetic strategies have been developed allowing for a certain control over the structure of the resulting polymer (linear, branched, crosslinked). Catalyzed and un-catalyzed polymerizations, monomer molar ratio, reaction time, and temperatures play an important role in the molecular weight and the branching or crosslinking of polymers. Elastomeric materials with low tensile strength (<1 MPa) and T_g_ values ranging from −10 to −50 °C have generally been obtained. When it comes to the molar ratio of monomers, the smaller the Gly/acid molar ratio, the bigger the number average molecular weight of the obtained polymer, independently of the diacid. The mechanical properties of poly(glycerol esters) can also be tuned to a large extent by the ratio of the monomers and the polymerization conditions, while the hydrophilic character can be adjusted through the synthetic procedure or through additional functionalization/modification steps. 

When it comes to catalysis, tin and titanium catalysts have been mostly employed, although many polymerizations are carried out without catalyst at higher temperatures. The inconvenient of metal-based catalysts is that traces of metal can potentially contaminate the produced polymer, making it improper for biomedical applications. On the other hand, enzymatic catalysis typically affords linear polyesters, as it has been exemplified by PGAd. Organocatalysis could solve some of these issues, although for the time being studies focus on ring-opening polymerizations. Additionally, the solvent selection is critical. Most polymerizations are carried out in bulk, but solvents are necessary for the processing of polymers. The use of benign solvents, such as acetic acid, should be further generalized, as it would afford greener polymers which are expected to be more cell-friendly. 

When glycerol is reacted with lactide or ε-caprolactone monomers, three-arm poly(glycerol lactide) and poly(glycerol ε-caprolactone) copolymers are obtained through ring-opening polymerization. Semicrystalline polymers with increasing molecular weight are afforded when the cyclic monomer/Gly ratio is increased. Besides glycerol, glycidol has proven to be a versatile and very useful starting block to prepare amorphous hyperbranched polyesters when combined with lactide or ε-caprolactone monomers, while using polyglycerol as starting material can lead to core-shell copolymers can be synthesized. 

Combining polymers with different characteristics yields more effective copolymers with improved properties. A wide range of random or block copolymers of glycerol and *α,ω*-diacids with other comonomers have been investigated, providing tailored hyperbranched polyesters for specific applications. In addition, acylated or ionic polyglycerol polyesters have been synthesized by post-polymerization modifications. Especially in the case of poly(glycerol sebacate) copolymers and nanocomposite materials were out of the scope of the present review article and have not been discussed. Post-synthetic modifications and copolymerizations are tools that should be exploited to further tune the properties glycerol polyesters, thus widening their application spectrum.

Due to their low cytotoxicity and interesting biodegradation profile, glycerol polyesters are particularly appropriate for drug delivery and tissue engineering applications. All synthesized polymers exhibited excellent in vitro and in vivo biocompatibility and most of these polyesters have been used for drug nanoencapsulation and controlled release formulations. Poly(glycerol sebacate), because of its similar mechanical properties with various soft tissues and an appropriate degradation timeframe, has been extensively studied for tissue engineering applications: bone and cartilage tissue, myocardial tissue, vascular tissue, etc. Human tissues are not homogenous, and now that the potential of PGSeb in this area has been demonstrated, the focus of research shifts towards the design and preparation of more complex scaffolds combining different mechanical properties and different cell types. 

To conclude this review article, hyperbranched polyesters of glycerol are a versatile class of biodegradable and biocompatible polymers, and the interest they have attracted is expected to substantially increase in the next decades. 

## Figures and Tables

**Table 1:** Experimental conditions for the polymerization of glycerol and succinic acid and properties of the resulting branched polymers.

Gly/SuA Molar Ratio	Experimental Conditions				M_n_ (g/mol)	T_g_ (°C)	Ref.
	Solvent	Catalyst	T (°C)	Time (h)			
1.5	-	-	190	24	844 ^e^, 868 ^f^	-	[14]
1.7	-	-	190	24	673 ^e^, 680 ^f^	-	[14]
1.9	-	-	190	24	606 ^e^, 623 ^f^	-	[14]
0.6	-	-	180	1.1	938 ^g^	−15.2	[16]
0.9	-	-	180	2.9	1011 ^g^	−14.8	[16]
1.2	-	-	180	5.3	754 ^g^	−29.9	[16]
0.5 ^a,b^	-	-	180	1.2	-	−10.9	[17]
0.8 ^a,c^	-	-	180	1.8	-	−2.7	[17]
1.3, 1.9 ^a,d^	-	-	180	1.5 or 6	-	16.6 or 33.4	[17]
1.0	Toluene	Dibutyl tin(IV) oxide	135	24	22,280 ^g^	-	[18]
0.5	Toluene	Dibutyl tin(IV) oxide	135	24	112,770 ^g^	-	[18]
1.0 ^h^	-	Titanium(IV) butoxide	initial 60 °C, final 150 °C	16 h in total	992 ^e^	-	[19]

^a^: mass ratio: g raw glycerol/g succinic acid; ^b^: pure glycerol; ^c^: refined glycerol (75% glycerol); ^d^: crude glycerol (15 or 30% glycerol); ^e^: determined by size exclusion chromatography; ^f^: determined by ^13^C NMR; ^g^: determined by gel permeation chromatography (GPC); ^h^: Pressure: 150 Pa.

**Table 2:** Experimental conditions for the polymerization of glycerol and glutaric acid and properties of the resulting branched polymers.

Gly/GA Molar Ratio	Experimental Conditions				M_n_ (g/mol)	DB (%)	T_g_ (°C)	Ref.
	Solvent	Catalyst	T (°C)	Time (h)				
1:2	DMF	Titanium(IV) butoxide	150	24	2402	54	-	[33]
1:2	DMF	Dibutyl tin(IV) oxide	150	10	1058	41	−50.1	[34]
1:2	Toluene	Dibutyl tin(IV) oxide	155	10	23,570	70.5	-	[18]
1:2	DMF	Dibutyl tin(IV) oxide	150	24	1474	45	-	[34]
1:2	Toluene	Dibutyl tin(IV) oxide	155	24	29,380	76.7	-	[18]
1:2 ^a^	Neat	Dibutyl tin(IV) oxide	initial 60 °C, final 150 °C	24 h in total	445,000	82	−31.3	[34]
1:1	Toluene	Dibutyl tin(IV) oxide	155	24	252,060	33.3	-	[18]

^a^: Pressure: 150 Pa.

**Table 3:** Experimental conditions for the polymerization of glycerol and adipic acid and properties of the resulting branched polymers.

Gly/AdA Molar Ratio	Experimental Conditions					M_w_ (g/mol)	T_g_ (°C)	Ref.
	Catalyst	T (°C)	Time (h)	Pressure	Thermal Curing (°C)			
1:1 ^b^	-	a) 150	17	atm	120	4500 ^a^	−15	[53]
		b) 150	4	0.5 mm Hg				
2:3 ^b^	-	a) 150	16	atm	120	12,500 ^a^	−2.5	[53]
		b) 150	0.5	0.5 mm Hg				
4:3	Dibutyltin oxide	140	11.5	atm	-	1250	-	[54]
1:1	Dibutyltin oxide	140	11.5	atm	-	14,000	-	[54]

^a^: prepolymer, before curing, ^b^: two-step reaction.

**Table 4:** Average molecular weights obtained for different experimental conditions.

Gly/AzA Molar Ratio	Experimental Conditions				M_n_ (g/mol)	Ref.
	Solvent	Catalyst	T (°C)	Time (h)		
1:1 ^a^	-	Titanium(IV) butoxide	initial 60 °C, final 150 °C	16 h in total	2316	[19]
1:1	-	-	140	14	1852	[75]
1:1	DMF or DMSO	Titanium(IV) butoxide	150	22	3245	[33]
1:1	Toluene	Dibutyltin(IV) oxide	135	24	4160	[18]
1:1.5	-	-	140	14	2873	[75]
1:1.6	-	-	140	14	2574	[75]
1:2	Toluene	Dibutyltin(IV) oxide	135	24	5410	[18]

^a^: Pressure: 150 Pa.

**Table 5:** Mechanical properties of PGSeb materials, prepared at 130 °C. Process A: the monomers were mixed at the defined Gly/SeA molar ratio. Process B: the monomers were mixed in a 1:1 Gly/SeA ratio, which was adjusted to 2:2.5 by adding SeA during curing.

Process	Gly/SeA Molar Ratio	Sol Content (%)	Tensile Strength (MPa)	Tensile Stress (MPa)	Elastic Modulus (MPa)	Elongation at Break (%)	Tensile Strain (%)	T_g_ (°C)	Water Contact Angle (°)	Ref.
A	2:2.5	75.13	0.210	-	0.07	114.5	-	−25.1	45.2	[97]
A	2:3	74.81	0.141	-	0.14	101.3	-	−27.1	47.9	[97]
A	2:3.5	69.23	0.699	-	1.45	57.2	-	−29.9	52.3	[97]
A	2:4	84.23	0.425	-	7.05	12.0	-	−32.2	56.6	[97]
B	2:2.5	61.87	-	0.61	0.55	-	236	−22.5	37.8	[91]
B ^a^	2:2.5	66.88	-	0.41	0.49	-	109	−26.1	43.4	[91]
B ^a^	2:2.5	62.75	-	0.64	0.45	-	179	−26.2	42.5	[91]

^a^: increased reaction time.

**Table 6:** Mecahnical properties and crosslinking density of PGSeb samples.

Gly/SeA Molar Ratio	Conditions		Ultimate Tensile Strength (MPa)	Young’s Modulus (MPa)	Rupture Strain (%)	Crosslinking Density (mol/m^3^)	Ref.
	Solvent	T (°C)					
2:2	Toluene	130		0.25		25.21 ± 8.84 ^a^	[89]
2:2.5	Toluene	130		0.57		56.73 ± 5.94 ^a^	[89]
2:3.3	Toluene	130		0.72		71.48 ± 8.84 ^a^	[89]
1:1	Neat	110	0.23	0.06	4.48	10 ± 4 ^b^	[101]
1:1	Neat	120	0.39	0.22	2.56	42 ± 17 ^b^	[101]
1:1	Neat	140	0.47	1.20	0.41	131 ± 50 ^b^	[101]

^a^: Calculated from Young, s modulus, E, according to n=E/3RT. R is the universal gas constant, and T is the temperature. b: Calculated with the Flory–Rehner expression for tetra-functional affine network, with Flory–Huggins parameter χ = 0.42.

**Table 7:** Properties of poly(L-lactide) polymers with a glycerol core.

Monomer/Gly Molar Ratio	Experimental Conditions				M_n_ (g/mol)	T_g_ (°C)	T_m_ (°C)	X (%)	Ref
	Catalyst	T (°C)	Time	Pressure					
3:1 ^a^	Sn(Oct)_2_	130	4 days		1400	−10	- ^d^		[177]
3:1 ^a^	TPhT	130	4 days		4300	−13	- ^d^		[177]
10:1 ^a^	Sn(Oct)_2_	130	4 days		5400	15	- ^d^		[177]
10:1 ^a^	TPhT	130	4 days		2200	14	- ^d^		[177]
20:1 ^b,c^	SnO	a) 140	8 h	4000 Pa	2600	6	- ^d^		[178]
		b) 160	8 h	70 Pa					
40:1 ^a^	Sn(Oct)_2_	130	4 days		9800	40	138	32.0	[177]
40:1 ^a^	TPhT	130	4 days		13,000	40	128	26.5	[177]
40:1 ^b,c^	SnO	a) 140	8 h	4000 Pa	3100	25	- ^d^		[178]
		b) 160	8 h	70 Pa					
80:1 ^b,c^	SnO	a) 140	8 h	4000 Pa	3300	37	98	16.6	[178]
		b) 160	8 h	70 Pa					
100:1 ^a^	Sn(Oct)_2_	130	4 days		14,000	47	158	58.9	[177]
100:1 ^a^	TPhT	130	4 days		43,000		170	55.8	[177]
100:1 ^b,c^	SnO	a) 140	8 h	4000 Pa	4700	41	119	17.7	[178]
		b) 160	8 h	70 Pa					
140:1 ^b,c^	SnO	a) 140	8 h	4000 Pa	3200	41	123	20.8	[178]
		b) 160	8 h	70 Pa					

^a^: L-lactide; ^b^: L-lactic acid; ^c^: two-step reaction, ^d^: not detected.

**Table 8:** Properties of PGCL synthesized under enzymatic or metallic catalysis (n.d.: not determined).

CL/Gly Molar Ratio	Experimental Conditions				M_n_ (g/mol)	T_m_ (°C)	X_c_^a^ (%)	Ref.
	Catalyst	Solvent	T (°C)	Time (h)				
1.5:1	Novozym 435	Toluene	70	4	1500	46	49	[215]
1.5:1	Novozym 435	Toluene	70	6	2400	48	51	[215]
9:1	Novozym 435	Toluene	70	4	2500	48	53	[215]
10:1	Novozym 435	Dioxane	70	4	2900	46	57	[214]
30:1	Novozym 435,	Dioxane	70	3	4900	53	68	[214]
10:1	Sn(oOct)_2_	-	130	48	6280	n.d.	n.d.	[203]
40:1	Sn(Oct)_2_	-	130	48	18,900	n.d.	n.d.	[203]
90:1	Sn(Oct)_2_	-	110	48	22,000	n.d.	n.d.	[206]
270:1	Sn(Oct)_2_	-	110	48	58,200	n.d.	n.d.	[206]

^a^: determined by DSC.

## Abbreviations

5-FU	5-fluorouracil
AdA	adipic acid
AzA	azelaic acid
BSA	bovine serum albumin
CAL	lipase B from *Candida Antarctica*
CIP	ciprofloxacin-HCl
CL	ε-caprolactone
Cyt-ara	cytosine arabinoside
DB	degree of branching
D-lact	D-lactide
DMF	N,N-dimethyl formamide
DMSO	dimethyl sulfoxide
DP	degree of polymerization
dPGS	dendritic polyglycerol sulfate
DSC	differential scanning calorimetry
DXM	dexamethasone
DXMP	dexamethasone phosphate
ECM	extracellular matrix
EDC	1-ethyl-(3,3-dimethylaminopropyl) carbodiimide
FA	folic acid
FDA	US Food and Drug Administration
FTIR	Fourier-transform infrared spectroscopy
Gld	glycidol
GA	glutaric acid
Gly	glycerol
GPC	gel permeation chromatography
h	hour
HA	hydroxyapatite
HBPG	hyperbranched polyglycerol
HBPG-NH_2_	amino hyperbranched polyglycerol
HPMA	N-(2-hydroxypropyl) methacrylamide
IBU-Na	ibuprofen sodium salt
Ind	indomethacin
IV	intrinsic viscosity
LaA	lactic acid
Lact	lactide
L-lact	L-lactide
L-leu	L-leucine
MG	monoglyceride
M_n_	number average molecular weight
MTX	methotrexate
M_w_	weight average molecular weight
MWD	molecular weight distributions
NHS	N-hydroxysuccinimide
NMR	nuclear magnetic resonance
NP	nanoparticle
OX26	transferrin antibody
PAMAM	polyamidoamine
PBS	phosphate buffer saline
PCL	poly(ε-caprolactone)
PDI	polydispersity index
PDL	pentadecalactone
PDLA	poly(D-lactic acid), poly(D-lactide)
PEG	polyethylene glycol
PEGDA	poly(ethylene glycol) diacrylate
PEI	polyethylenimine
PEO	poly(ethylene oxide)
PG	polyglycidol
PGA	poly(glycolic acid), poly(glycolide)
PGAd	poly(glycerol adipate)
PGAdS	stearic acid-modified PGAd
PGAz	poly(glycerol azelate)
PGCL	poly(glycerol ε-caprolactone)
PGGlu	poly(glycerol glutarate)
PGL	poly(glycerol lactide)
PGly	poly(glycerol)
PGSeb	poly(glycerol sebacate)
PGSMA	poly(glycerol succinate-co-maleate)
PGSub	poly(glycerol suberate)
PGSuc	poly(glycerol succinate)
PHA	poly(hydroxyalkanoates)
PLA	poly(lactic acid), poly(lactide)
PLGA	poly(lactic-co-glycolic acid), poly(lactide-co-glycolie)
PLLA	poly(L-lactic acid), poly(L-lactide)
PNIPAAm	poly(N-isopropylacrylamide)
PTX	paclitaxel
PVA	poly(vinyl alcohol)
RBITC	Rhodamine B Isothiocyanate
ROP	ring-opening-polymerization
RPC	retinal progenitor cells
SeA	sebacic acid
SEC	size exclusion chromatography
SEM	scanning electron microscopy
Sn(Oct)_2_	tin(II) 2-ethylhexanoate, stannous octoate
SuA	succinic acid
TAC	tacrolimus
T_g_	glass transition temperature
THF	tetrahydrofurane
Tm	melting temperature
TPT	tetraphenyl tin
TSA	p-toluenesulfonic acid
